# Urine Sample-Derived Cerebral Organoids Suitable for Studying Neurodevelopment and Pharmacological Responses

**DOI:** 10.3389/fcell.2020.00304

**Published:** 2020-05-14

**Authors:** Victor J. T. Lin, Jiangnan Hu, Ashwini Zolekar, Liang-Jun Yan, Yu-Chieh Wang

**Affiliations:** Department of Pharmaceutical Sciences, UNT System College of Pharmacy, University of North Texas Health Science Center, Fort Worth, TX, United States

**Keywords:** urinary epithelial cells, hiPSCs, hESCs, neurodevelopment, cerebral organoids

## Abstract

Cerebral organoids (COs) developed from human induced pluripotent stem cells (hiPSCs) have been noticed for their potential in research and clinical applications. While skin fibroblast-derived hiPSCs are proficient at forming COs, the cellular and molecular features of COs developed using hiPSCs generated from other somatic cells have not been systematically examined. Urinary epithelial cells (UECs) isolated from human urine samples are somatic cells that can be non-invasively collected from most individuals. In this work, we streamlined the production of COs using hiPSCs reprogrammed from urine sample-derived UECs. UEC-derived hiPSC-developed COs presented a robust capacity for neurogenesis and astrogliogenesis. Although UEC-derived hiPSCs required specific protocol optimization to properly form COs, the cellular and transcriptomic features of COs developed from UEC-derived hiPSCs were comparable to those of COs developed from embryonic stem cells. UEC-derived hiPSC-developed COs that were initially committed to forebrain development showed cellular plasticity to transition between prosencephalic and rhombencephalic fates *in vitro* and *in vivo*, indicating their potential to develop into the cell components of various brain regions. The opposite regulation of AKT activity and neural differentiation was found in these COs treated with AKT and PTEN inhibitors. Overall, our data reveal the suitability, advantage, and possible limitations of human urine sample-derived COs for studying neurodevelopment and pharmacological responses.

## Introduction

Recent progress in stem cell research and culture techniques has enabled human organoid models that better mimic the development, structure, and function of human organs (Huch et al., 2017; Rossi et al., 2018; Takahashi, 2019). In particular, human cerebral organoids (COs) offer exciting opportunities to mechanistically investigate brain development and pathogenesis in human cell-based, self-organized, and defined conditions (Lancaster et al., 2013; Quadrato and Arlotta, 2017; Arlotta, 2018).

Human pluripotent stem cells (hPSCs), including human embryonic stem cells (hESCs) and induced pluripotent stem cells (hiPSCs), are often used as starting materials to generate COs. Many recent studies have leveraged COs and made important discoveries regarding developmental biology, disease mechanisms, and pharmacological responses in the central nervous system (Lancaster et al., 2013; Pasca et al., 2015; Bershteyn et al., 2017; Dakic et al., 2017; Lee et al., 2017; Quadrato et al., 2017; Watanabe et al., 2017; Trujillo and Muotri, 2018; Kanton et al., 2019; Pollen et al., 2019; Velasco et al., 2019; Zhang et al., 2019). Transcriptomic profiling has revealed that cortical cells generated in human COs use gene expression programs highly similar to those of the human fetal neocortex to organize into cerebral cortex-like regions (Camp et al., 2015). This similarity highlights the suitability of COs for studying the molecular and cellular features of human cortical development at an early stage. Although hiPSCs reprogrammed from dermal fibroblasts can develop into COs as revealed by many studies (Lancaster et al., 2013; Bershteyn et al., 2017; Velasco et al., 2019; Xu et al., 2019; Zhang et al., 2019), the capacity of hiPSCs reprogrammed from other somatic cells to generate COs has not been systematically characterized.

Many types of somatic cells, including dermal fibroblasts, peripheral blood mononuclear cells, hair follicle keratinocytes, and urinary epithelial cells (UECs), have been used in cell reprogramming to acquire hiPSCs (Takahashi et al., 2007; Zhou et al., 2012; Streckfuss-Bomeke et al., 2013; Wen et al., 2016). Among them, UECs can be harvested from an individual’s urine samples that represent a non-invasive source for obtaining human somatic cells. A major advantage of using urine samples to acquire UECs for cell reprogramming is that procedures for urine collection could be easily applied to virtually all individuals and may be performed by personnel without advanced training. In effect, harvesting somatic cells from this sample source can be achieved in any cohort with minimal concerns. Thus, COs generated using urine sample-derived hiPSCs would be a highly useful platform for studying human disease such as genetic disorders that involve brain developmental defects.

Here, we streamlined the production of COs using hiPSCs reprogrammed from urine sample-derived UECs as well as characterized the cellular and molecular features of these COs during development and in response to the treatment of small molecules. Our results indicate that urine samples are highly practical biospecimens to enable the generation of personalized COs. Viable and reprogrammable human UECs can be readily isolated using centrifugation-based and filtration-based methods. COs developed from the UEC-derived hiPSCs with a normal karyotype highly resemble COs developed from normal hESCs that have shown a robust capacity for neurogenesis and CO formation in this and previous studies (Lancaster et al., 2013; Renner et al., 2017). In addition, COs that were developed from the UEC-derived hiPSCs and initially committed to forebrain development present cellular plasticity for the induced transition from a prosencephalic fate into a mesencephalic or rhombencephalic fate. Upon transplantation into the mouse brain, the COs develop distinctively in response to the different transplanted regions.

Although UEC-derived hiPSCs may have intrinsic features that require specific protocol optimization for the successful production of COs, UEC-derived hiPSC-developed COs are similar to hESC-developed COs at cellular and molecular levels. The versatility of urine sample-derived COs as a useful model for the investigation of neurodevelopment and pharmacological responses *in vitro* and *in vivo* is also revealed in our work.

## Results

### Urine Sample-Derived hiPSCs With Cellular and Molecular Features Similar to WA09 hESCs

**FIGURE 1:**
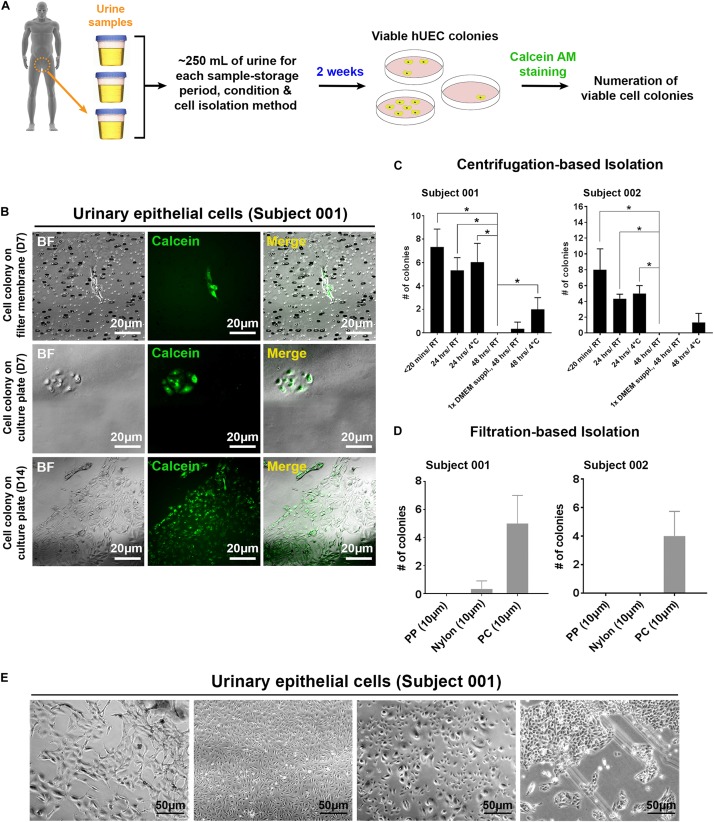
The isolation of proliferative UECs from human urine samples by centrifugation and filtering approaches. **(A)** A schematic illustration of the procedure to isolate UECs from human urine samples. **(B)** Proliferative UECs isolated from two human subjects. **(C)** The efficiencies of UEC isolation in urine samples from the two subjects preserved under the indicated conditions prior to centrifugation (mean ± SD; *n* = 3, *p < 0.05, *t*-test). 1× DMEM suppl: 10× DMEM spiked into urine sample at the final concentration of 10%. **(D)** The efficiencies of UEC isolation in urine samples from the two subjects using the filtration-based approach with filter membranes (pore size: 10 μm) made of distinct materials (mean ± SD; *n* = 3). PP: polypropylene. PC: polycarbonate. **(E)** The morphological features of different UEC subpopulations isolated from the subject 001.

In contrast to fibroblasts isolated from skin-biopsy samples, UECs isolated from human urine samples can be readily obtained from a completely non-invasive procedure. From the collection of 200–400 ml urine from each individual (Figure 1A), we established primary cultures of UECs from different human subjects. Viable UECs (Figure 1B) can be obtained from the samples through either centrifugation-based or filtration-based isolation. To gauge how long a urine sample may be preserved without affecting the viability of isolated UECs, we tested cell isolation in urine samples stored under various conditions. As expected, the freshly collected samples (samples subjected to cell isolation within 20 min of post-collection) gave rise to the most viable, proliferative UEC colonies in culture. The prolonged storage of urine samples at room temperature largely diminished the viability of isolated UECs (Figure 1C). Although the number of cell colonies from the urine kept at 4°C was also reduced due to prolonged storage, the low-temperature condition partially preserved the viability of UECs in urine up to 48 h (Figure 1C). While failing to obtain any colony from urine samples stored for 72 h in all the tested conditions, our results indicate the feasibility of applying this sample-collection method to harvesting viable cells from individuals in different geographic locations that require a short period of sample storage and/or transportation prior to cell isolation. For filtration-based isolation, we filtered urine samples using sterilized membranes made of different materials. By directly culturing cells that remained on the filter membranes, the isolation of proliferative UECs was achievable using polycarbonate (PC) membranes with a pore size of 10 μm (Figure 1D).

**FIGURE 2:**
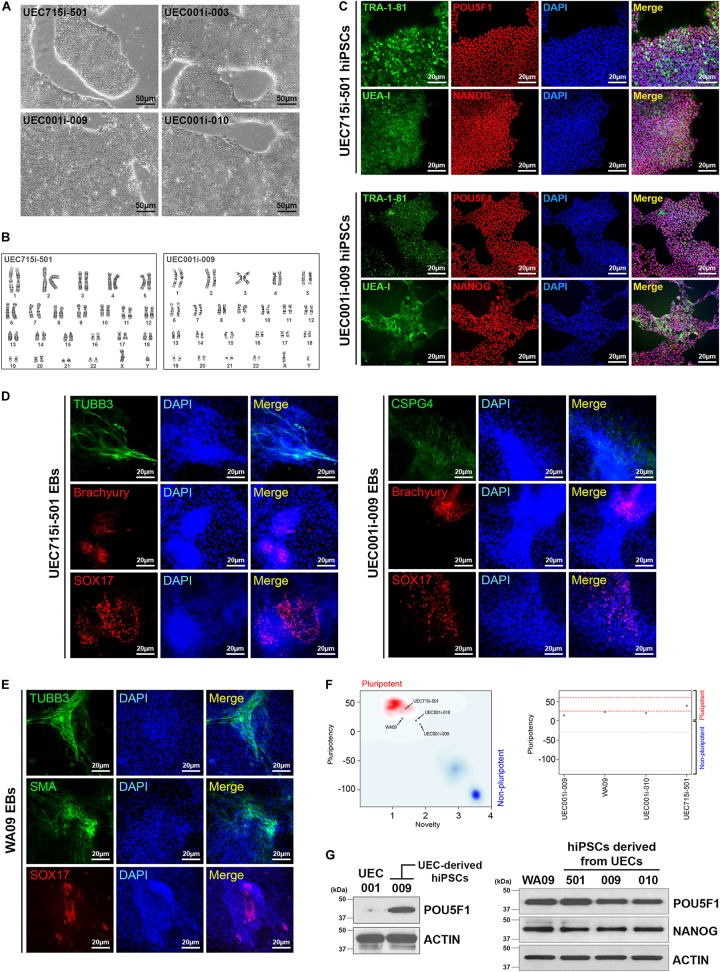
Normal hiPSCs established by cell reprogramming in UECs isolated from urine samples. **(A)** The pluripotent cell morphology of UEC-derived hiPSCs. UEC715i-501 hiPSCs were generated using a Sendai virus-mediated, non-integrative reprogramming method. UEC001i-003, UEC001i-009, and UEC001i-010 hiPSCs were generated using retrovirus-mediated delivery of expression vectors for transcription factors POU5F1, SOX2, KLF4, and MYC. **(B)** UEC715i-501 and UEC001i-009 hiPSCs (passage numbers 19 and 16, respectively) showed apparently normal karyotypes (46, XY). **(C)** The UEC-derived hiPSCs were positive of cellular pluripotency markers TRA-1-81, UEA-I, POU5F1, and NANOG. **(D)** UEC-derived hiPSCs formed embryoid bodies (EBs) containing cells relevant to all three germ-layer lineages. TUBB3 and CSPG4: ectoderm. Brachyury: mesoderm. SOX17: endoderm. **(E)** WA09 hESCs used in this study also formed EBs containing cells relevant to all three germ-layer lineages. TUBB3 and CSPG4: ectoderm. SMA: mesoderm. SOX17: endoderm. **(F)** The Pluritest results of undifferentiated WA09 hESCs and UEC-derived hiPSCs revealed that their transcriptomic features are highly similar to those of the pluripotent samples included in the Pluritest database. **(G)** POU5F1 and NANOG were expressed similarly in undifferentiated WA09, UEC715i-501, UEC001i-003, UEC001i-009, UEC001i-010 cells. UEC001: proliferative UECs isolated from the subject 001.

Morphological heterogeneity was frequently seen in cells isolated from urine. This heterogeneity was observed even in the cells derived from the same individual (Figure 1E), indicating that distinct cell types may exist in each collection of urine samples. Although we cannot pinpoint which type(s) of cells from each urine sample was reprogrammed and gave rise to hiPSCs, regardless of the UEC heterogeneity, we have obtained hPSC-like cells from four different individuals by cell reprogramming with retrovirus-mediated delivery of POU5F1, SOX2, KLF4, and MYC. Similar to WA09 hESCs and UEC715i-501 hiPSCs that were generated by Sendai virus-mediated reprogramming in the UECs from another individual, the feeder-free cultures of UEC001i-009 and UEC001i-010 hiPSCs had typical hPSC morphology (Figure 2A) and a normal karyotype (Figure 2B). These UEC-derived hiPSCs expressed multiple biomarkers for cellular pluripotency (Figure 2C). Like WA09 hESCs, the UEC-derived hiPSCs formed embryoid bodies (EBs) that contain cells belonging to three-germ-layer lineages (Figures 2D,E). When they were examined by the PluriTest (Muller et al., 2011), similar to WA09 hESCs, all the tested UEC-derived hiPSCs showed transcriptomic features unique to other *bona fide* hPSCs samples (Figure 2F). The expression levels of POU5F1 and NANOG in the UEC-derived hiPSCs were comparable to those in WA09 hESCs (Figure 2G). These data demonstrated that, regardless of cell reprogramming methods, hiPSCs similar to WA09 hESCs can be generated using UECs collected from different individuals.

### COs Developed From Urine Sample-Derived hiPSCs

WA09 hESC-derived COs have been used in several studies (Lancaster et al., 2013; Camp et al., 2015; Renner et al., 2017; Watanabe et al., 2017; Matsui et al., 2018; Kanton et al., 2019). Because the capacity of WA09 hESCs to form COs was previously confirmed and characterized, we used WA09 hESCs as a standard to compare against UEC-derived hiPSCs for organoid formation.

**FIGURE 3:**
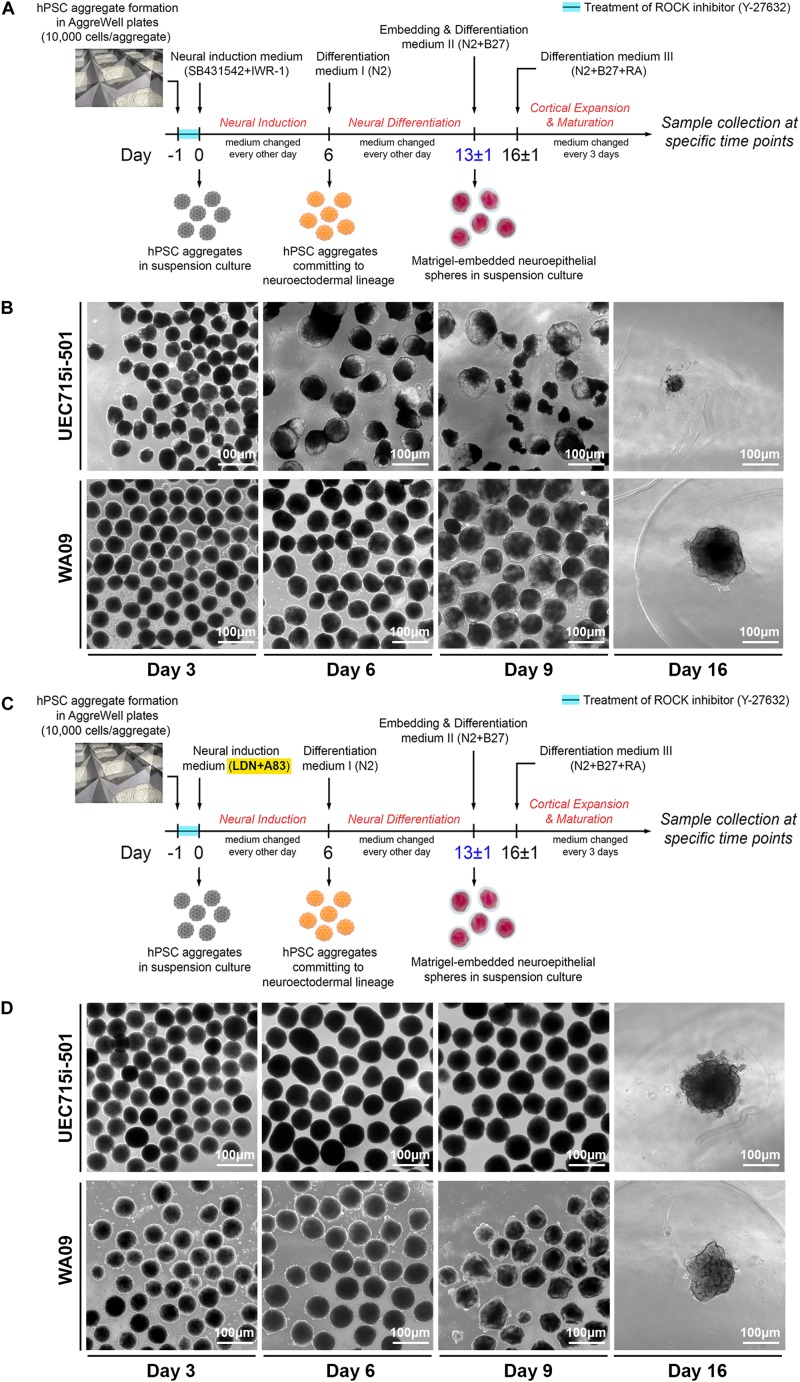
The differential development of COs from UEC-derived hiPSCs and WA09 hESCs. **(A)** A schematic illustration of the procedure that generated COs from the hiPSCs and hESCs by inhibition of TGFβ and WNT signaling to induce their neuroectodermal commitment. **(B)** The morphological representations at the indicated time points of the COs that were developed in response to inhibition of TGFβ and WNT signaling. Within 2 weeks after the induction of neuroectodermal commitment, the disintegration was observed in most organoids developed from UEC715i-501 hiPSCs. **(C)** A schematic illustration of the procedure that generated COs from the hiPSCs and hESCs by inhibition of TGFβ and BMP signaling to induce their neuroectodermal commitment. **(D)** The morphological representations at the indicated time points of the COs that were developed in response to inhibition of TGFβ and BMP signaling. Two weeks after the induction of neuroectodermal commitment, most organoids developed from UEC715i-501 hiPSCs remained intact and continued to grow.

Based on the protocol illustrated in Figure 3A, we reproducibly developed COs from undifferentiated WA09 hESCs. Despite minor variations, most WA09 cell aggregates gave rise to compact spheroids (Figure 3B) in response to suspension culture for 6 days in an FGF2-free neural induction medium that contains 10 μM SB431542 (a TGFβ receptor inhibitor) and 3 μM IWR-1-endo (an AXIN1/2 stabilizer). The structure of these cell spheroids often remained densely packed with a clear and smooth border before their transition into the N2-containing differentiation medium I for an additional culture period of 6–7 days, where the cells were further committed to the neuroectoderm lineage and continued developing into healthy spheres with translucent outer layers of neuroepithelial cells prior to matrigel embedding (Figure 3B). Most of the WA09 neuroepithelial spheres embedded in matrigel droplets kept developing in the N2/B27-containing differentiation medium II and retinoic acid (RA)-containing differentiation medium III to allow neurogenesis, gliogenesis, and neural cell maturation. Along this process, the neuroepithelial cell layers continued to enlarge, invaginate, and form crest-like structures. Similar morphological changes were observed during CO development initiated using the neural induction medium that contains 0.1 μM LDN193189 (a BMP receptor inhibitor) and 0.5 μM A83-01 (an ALK4/5/7 inhibitor) for dual SMAD inhibition in WA09 hESC aggregates (Figures 3C,D).

While the aggregates of UEC001i-009, UEC001i-010, and UEC715i-501 hiPSCs developed into multicellular structures that were morphologically similar to the WA09 neuroepithelial spheres (Figure 3D and Supplementary Figure S1) in the LDN193189/A83-01-containing induction medium, most UEC-derived hiPSC aggregates cultured in the SB431542/IWR-1-containing induction medium developed into neuroepithelial spheres with poor structural stability (Figure 3B and Supplementary Figure S1). When matrigel embedding was attempted with the defective spheres, they often failed to continuously develop (Figure 3B). These findings suggest that, though capable of CO formation, UEC-derived hiPSCs appear to have unique intrinsic features that require specific treatment for the successful development of COs.

### Neural Cells in COs Developed From Urine Sample-Derived hiPSCs

**FIGURE 4:**
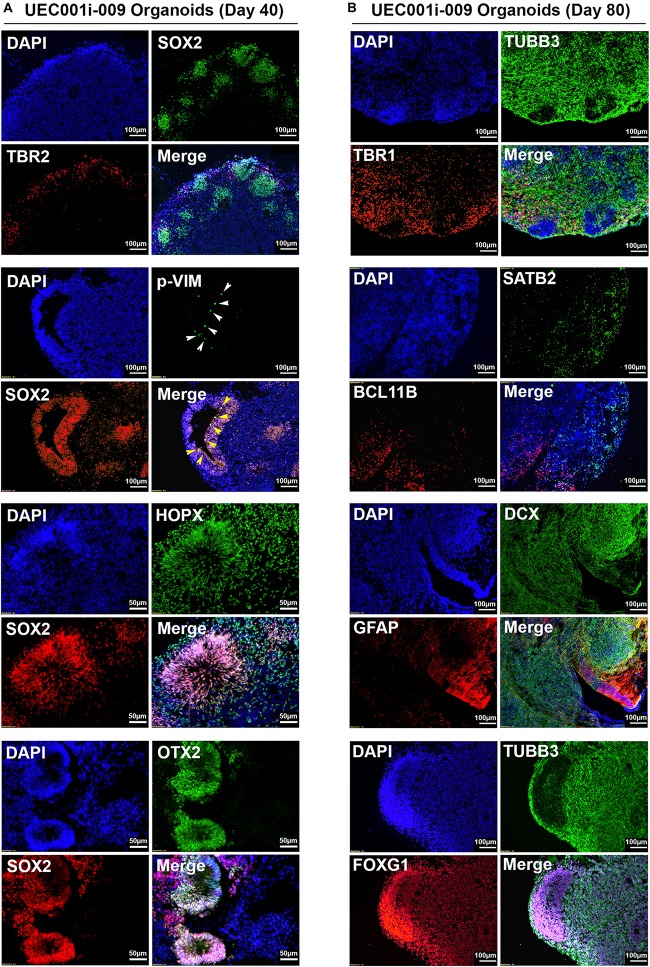
Neural lineage markers were expressed by the cells in COs developed from UEC-derived hiPSCs. **(A)** The expression of multiple markers for neural progenitor cells in the COs collected at day 40 of development. **(B)** The expression of multiple markers for neuronal and astroglial cells in the COs collected at day 80 of development. The development of these CO samples was based on the procedure that inhibited TGFβ and BMP signaling to induce neuroectodermal commitment.

As shown in Figure 4, multiple types of cells indicated by the expression of distinct markers were found in COs developed from the UEC-derived hiPSCs. The major cell types found in these COs included cells that express neural progenitor markers (SOX2, p-VIM, TBR2, OTX2, and HOPX), neuronal markers (TUBB3, DCX, TBR1, BCL11B, and SATB2), and an astroglial marker (GFAP). Different types of cells showed their unique distribution and organization that resemble specific layers and anatomical structures, which can be seen in the developing brain. In addition, the pervasive presence of FOXG1-positive cells in these COs indicated that they were committed to the development of the telencephalon-like tissue. By monitoring the presence of TUBB3 + and GFAP + cells in the samples collected at different time points (Supplementary Figure S2), we confirmed that astrogliogenesis followed neurogenesis in the COs developed from UEC-derived hiPSCs, resembling the temporal sequence of normal cortical development in mammals (Sauvageot and Stiles, 2002).

**FIGURE 5:**
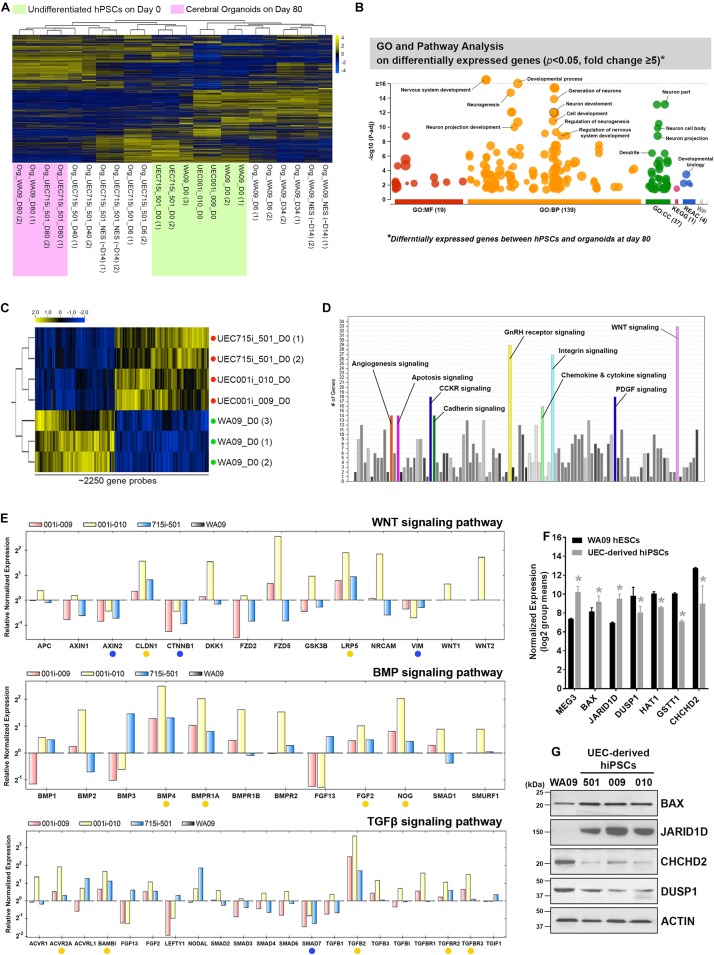
The molecular features of gene expression in COs developed from UEC-derived hiPSCs. **(A)** The heatmap representation of non-supervised clustering based on the global gene-expression profiles in the hPSC and their CO samples. The gene expression profiles in the organoids developed for 80 days from UEC715i-501 hiPSCs and WA09 hESCs were clustered together and segregated away from others, supporting the molecular similarity of COs developed from UEC-derived hiPSCs and WA09 hESCs. **(B)** The result of gene ontology and pathway analysis of differentially expressed genes identified through comparing the samples of undifferentiated WA09 hESCs and UEC715i-501 hiPSCs with the day-80 samples of COs developed from these hPSCs. The differentially expressed genes were found significantly involved in the biological processes of development and neurogenesis. **(C)** The heatmap representation of ∼2250 probes that measured the relative expression levels of differentially expressed genes (*p* < 0.05, *t*-test) in the UEC-derived hiPSC and WA09 hESC samples without differentiation. *Red dots:* UEC-derived hiPSCs. *Green dots:* WA09 hESCs. **(D)** Gene ontology and pathway analysis revealed the signaling pathways that are highly relevant to the differentially expressed genes identified between the sample groups of undifferentiated UEC-derived hiPSCs and WA09 hESCs. **(E)** The expression of the selected genes relevant to the regulation of WNT, BMP, and TGFβ signaling was examined using qRT-PCR array analysis. *Yellow dots:* genes hyperexpressed in UEC-derived hiPSCs. *Blue dots:* genes hypoexpressed in UEC-derived hiPSCs. **(F)** The expression of representative genes that are differentially expressed in UEC-derived hiPSCs and WA09 hESCs as well as involved in the regulation of cellular responses to stimuli, epigenetic control, or protein posttranslational modifications (mean ± SD; *n* = 4 for hiPSC samples, *n* = 3 for hESC samples, *p < 0.05, *t*-test), according to gene expression array analysis. **(G)** The protein expression of the selected genes detected by western blotting confirmed their differential expression in UEC-derived hiPSCs and WA09 hESCs.

Array-based transcriptomic profiling followed by non-supervised clustering analysis revealed that, despite some variations in COs formed by UEC-derived hiPSCs and WA09 hESCs at the early stage (day 6 to day 40) of development, the gene expression profiles of the hESC- and hiPSC-developed COs were highly similar after 80 days of development and segregated away from those of the undifferentiated hPSCs (Figure 5A). Gene ontology and pathway analysis on the differentially expressed genes (*p* < 0.05, fold change ≥ 5) in day-80 COs and undifferentiated hPSCs indicated that these differentially expressed genes are strongly relevant to the biological process and signaling pathways of nervous system development, neurogenesis, and neuron projection development (Figure 5B). These molecular features further highlight the neural lineage and similarity of COs developed from UEC-derived hiPSCs and WA09 hESCs.

### Gene-Expression Features Associated With the Distinct Responses of UEC-Derived hiPSCs and WA09 hESCs in Neural Induction for CO Development

Compared with undifferentiated WA09 hESCs, undifferentiated UEC-derived hiPSCs appeared to present a distinct expression pattern of certain genes (Figure 5A and Supplementary Figure S3A). The differential expression of a panel of selected genes in three UEC-derived hiPSC lines and WA09 hESCs were confirmed by qRT-PCR (Supplementary Figure S3B). It is possible that the genes that are intrinsically hyper- or hypo-expressed in the UEC-derived hiPSCs may contribute to their preference for the LDN193189/A83-01-containing induction medium in forming COs (Figure 3).

Through differential expression analysis (*p* < 0.05) between WA09 hESC and UEC-derived hiPSC samples, around 2250 gene probes were identified (Figure 5C). Gene ontology and pathway analysis revealed that multiple signaling pathways, including WNT signaling, gonadotropin-releasing hormone (GnRH) receptor signaling, and integrin signaling pathways, are enriched by these differentially expressed genes (Figure 5D). Because WNT, BMP, and TGFβ signaling pathways have frequent crosstalk and are highly relevant to the regulation of neurodevelopment and morphogenesis (Munji et al., 2011; Bond et al., 2012; Dias et al., 2014), we examined the expression of selected genes involved in these signaling pathways using qRT-PCR arrays. Compared with the gene expression in WA09 hESCs, multiple genes, including *AXIN2*, *CLDN1*, *CTNNB1*, *LRP5*, *VIM*, *BMP4*, *BMPR1A*, *FGF2*, *NOG*, *ACVR2A*, *BAMBI*, *SMAD7*, *TGFB2*, *TGFBR2*, *TGFBR3*, were either hypoexpressed or hyperexpressed consistently in the UEC-derived hiPSC lines that were analyzed (Figure 5E). In addition, we found the hyperexpression of the *MEG3*, *BAX*, *JARID1D* genes and the hypoexpression of the *DUSP1*, *HAT1*, *GSTT1*, and *CHCHD2* genes in the UEC-derived hiPSCs (Figures 5F,G).

**FIGURE 6:**
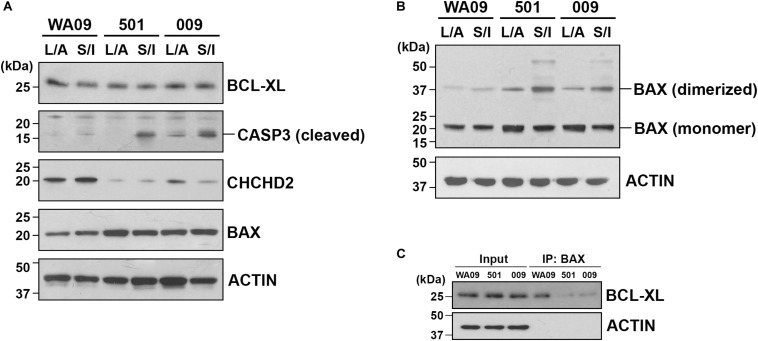
The expression of CHCHD2, the dimerization of BAX, and the interaction between BCL-XL and BAX in the aggregates of UEC-derived hiPSCs and WA09 hESCs that were cultured in LDN193189/A83-01-containing and SB431542/IWR-1-containing media. **(A)** The expression and activation of CHCHD2, BAX, BCL-XL, and CASP3 were revealed by western blotting in the indicated cell aggregate samples that were cultured in the indicated media. **(B)** Dimerized BAX (∼40 kDa) with electromobility shift from BAX monomer (∼20 kDa) was detected by western blotting in the cell aggregate samples. **(C)** BCL-XL interacted and co-immunoprecipitated with BAX in the cell aggregate samples cultured in the SB431542/IWR-1-containing medium was detected by western blotting. 501: UEC715i-501 hiPSCs. 009: UEC001i-009 hiPSCs. L/A: LDN193189/A83-01-containing medium. S/I: SB431542/IWR-1-containing medium. All the cell aggregates were cultured in the indicated media for 6 days prior to sample collection and analysis.

CHCHD2 is a mitochondria-associated protein that can function as an apoptosis inhibitor by promoting the interaction of BCL-XL and BAX and limiting the activation of BAX (Liu et al., 2015). Having the hypoexpression of the *CHCHD2* gene and the hyperexpression of the *BAX* gene identified in the UEC-derived hiPSCs, we examined whether the low expression of CHCHD2 and the activation of BAX may be involved in the challenge of CO development based on SB431542/IWR-1-mediated neural induction in the UEC-derived hiPSCs. Cell aggregates cultured parallelly in the LDN193189/A83-01-containing and SB431542/IWR-1-containing induction media were compared. Indicated by cleaved CASP3, enhanced apoptosis occurred in the aggregates of UEC001i-009 and UEC715i-501 hiPSCs, but not in the aggregates of WA09 hESCs, that were cultured in the SB431542/IWR-1-containing medium (Figure 6A). BAX multimerization was also enhanced in the aggregates of the UEC-derived hiPSCs (Figure 6B). While BCL-XL was similarly expressed in the aggregates of the UEC-derived hiPSCs and WA09 hESCs (Figure 6A), reduced interaction between BAX and BCL-XL was found in the aggregates of the UEC-derived hiPSCs (Figure 6C), in parallel with their hypoexpression of CHCHD2 (Figure 6A).

These findings support that UEC-derived hiPSCs and WA09 hESCs have intrinsic differences in signaling networks likely to affect responses to growth factors, neurogenesis, apoptotic propensity, and CO formation.

### Cellular Plasticity That Permits Cell-Fate Conversion in COs Developed by UEC-Derived hiPSCs

Based on a published protocol (Muguruma et al., 2015), we generated cerebellar organoids from UEC-derived hiPSCs and analyzed their gene expression. These cerebellar organoids showed the downregulation of a forebrain marker (FOXG1) and the upregulation of hindbrain markers (GBX2, HOXA2, and HOXB4) during development (Supplementary Figure S4A), suggesting that cell signaling for the development of the hindbrain tissue in response to patterning stimuli properly functions in the UEC-derived hiPSCs.

**FIGURE 7:**
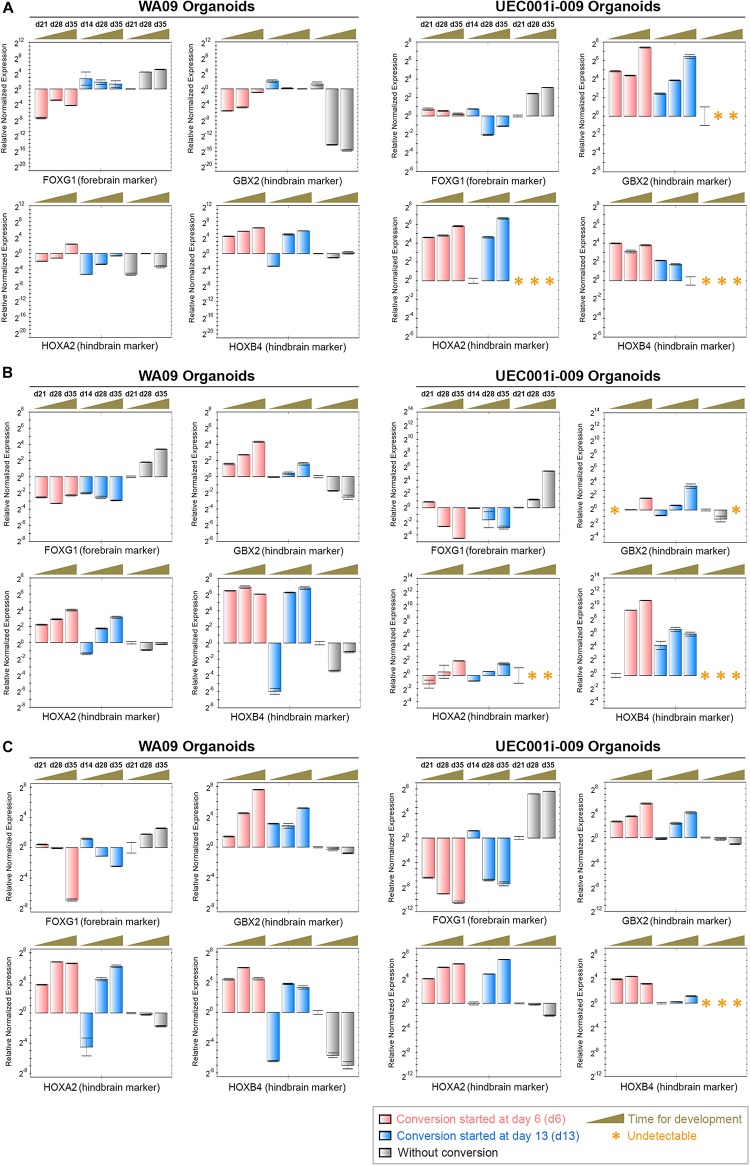
The gene expression alterations of forebrain and hindbrain markers due to the exposure of COs to FGF2, FGF19, and CHIR99021 (a WNT-signaling activator). **(A)** The expression of the *FOXG1*, *GBX2*, *HOXA2*, and *HOXB4* genes (marker genes) was determined by qRT-PCR analysis in the indicated organoid samples (*n* = 3 at each indicated time point for sample collection) that were developed through the procedure of cerebral induction 1 with and without the conversion treatment depicted in Supplementary Figure S4. The upregulation of FOXG1 as well as the suppression of GBX2, HOXA2, and HOXB4 in COs were reversed in the COs with rhombencephalic conversion mediated by the treatment of FGF2, FGF19, and CHIR99021. **(B)** The expression of the marker genes was determined by qRT-PCR analysis in the indicated organoid samples (*n* = 3 at each indicated time point for sample collection) that were developed through the procedure of cerebral induction 2 with and without the conversion treatment depicted in Supplementary Figure S4. The upregulation of FOXG1 as well as the suppression of GBX2, HOXA2, and HOXB4 in COs were reversed in the COs with the conversion treatment. **(C)** The expression of the marker genes was determined by qRT-PCR analysis in the indicated organoid samples (*n* = 3 at each indicated time point for sample collection) that were developed through the procedure of cerebral induction 3 with and without the conversion treatment depicted in Supplementary Figure S4. The upregulation of FOXG1 as well as the suppression of GBX2, HOXA2, and HOXB4 in COs were reversed in the COs with the conversion treatment. Time points for sample collection relevant to each experimental setting are presented in the days (d) of development. All data represent mean ± SD.

Having the capacity of forming cerebellar organoids determined in UEC-derived hiPSCs, we subsequently tested cellular plasticity in COs through potential conversion from their prosencephalic fate into a rhombencephalic fate. COs formed by UEC-derived hiPSCs and WA09 hESCs in response to three methods for cerebral patterning (Supplementary Figure S4B) consistently showed the upregulation of FOXG1 during the initial 35 days of development (Figure 7). In addition, the repression of GBX2, HOXA2 and HOXB4 was found in these COs (Figure 7). These findings were expected and relevant to the normal prosencephalic development of COs. After being transferred into the conversion media that contain SB431542, FGF2, and FGF19, the organoids with conversion started during the initial 14 days of development (Supplementary Figure S4B) showed downregulated FOXG1 accompanied by the upregulation of GBX2, HOXA2, and HOXB4, independent of hPSC sources and the methods for their initial cerebral patterning (Figure 7). These findings indicate that, like hESC-developed COs, COs developed from UEC-derived hiPSCs possess cellular plasticity that permits conversion between prosencephalic and rhombencephalic fates.

### The Distinct Response of COs Formed by UEC-Derived hiPSCs to Different Transplanted Regions in the Mouse Brain

**FIGURE 8:**
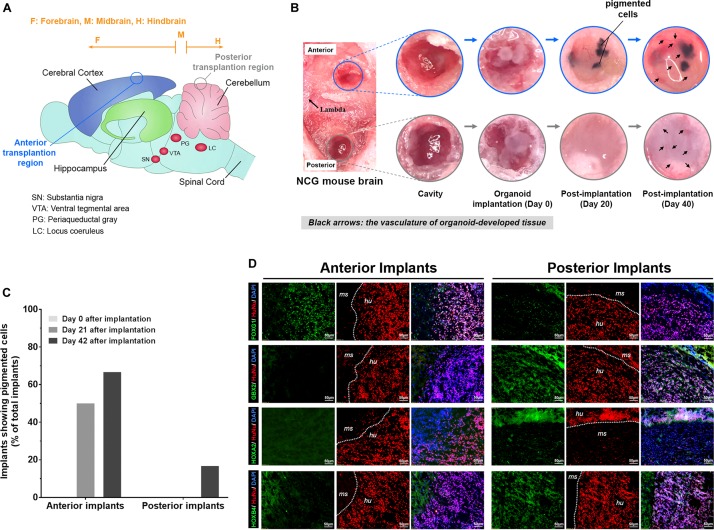
The transplantation of COs developed from UEC-derived hiPSCs into the cerebral cortex and cerebellum of the mouse brain led to the continuation of distinctive neural development in the organoids at these transplantation sites. **(A)** The schematic illustration of regions in the mouse brain for the anterior and posterior transplantation of the COs developed from UEC001i-009 hiPSCs. **(B)** The continued development of the implanted COs was monitored periodically through a cranial window under a surgical stereomicroscope. The vascularization of the tissue formed by the implanted COs was clearly visible at day 40 of post-implantation. **(C)** The percentage of the implanted organoids (*n* = 6) that developed into tissue with pigmented cells. **(D)** In contrast to the anterior implants (i.e., the tissue formed by the COs transplanted in the cerebral cortex of the mouse brain), the posterior implants (i.e., the tissue formed by the COs transplanted in the cerebellum of the mouse brain) presented with cells that showed the lower expression of FOXG1 and the higher expression of GBX2, HOXA2, and HOXB4 detected by immunofluorescence staining. HuNu: human nuclear antigen. ms: mouse tissue. hu: human tissue. The brain samples with organoid implants were harvested at day 42 of post-implantation.

To study the continued development of COs formed by UEC-derived hiPSCs *in vivo*, the organoids that went through the initial 14 days of *in vitro* development were transplanted to the anterior and posterior brain regions of adult mice with severe combined immunodeficiency (*n* = 6). Independent of the transplanted regions (Figure 8A), the CO-developed tissue was vascularized (Figure 8B), indicating that the transplanted COs survived and continued to develop in the animal brain. Three weeks after implantation, three anterior implants developed visible pigmented areas (Figure 8B). In contrast, pigmented cells were not observed in any implant in the posterior brain regions of the same mice (Figure 8C). By the end of 6 weeks after implantation, pigmented areas were visible in four anterior and one posterior implants (Figure 8C). The distinct frequencies of pigmented cells present in the anterior and posterior implants suggest that the microenvironments of different brain regions may substantially affect the continued development of the transplanted COs. Immunofluorescence staining revealed that, compared with the anterior implants, the posterior implants contained cells with the hypoexpression of FOXG1 and the upregulation of GBX2, HOXA2, and HOXB4 (Figure 8D). Our findings reveal that conversion from a prosencephalic fate to a rhombencephalic fate in COs developed by UEC-derived hiPSCs is likely to occur *in vivo* and be driven by the microenvironment and niche signaling in the host’s hindbrain tissue.

### The Opposite Responses of AKT Signaling and Neural Differentiation in COs Developed From UEC-Derived hiPSCs Treated With AKT and PTEN Inhibitors

**FIGURE 9:**
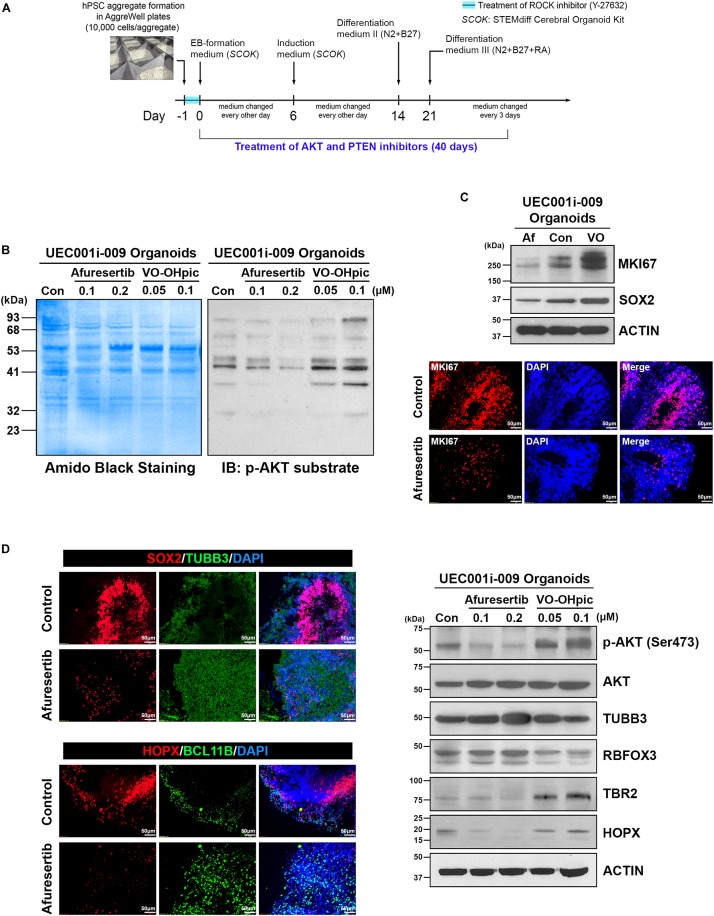
AKT and PTEN inhibitors led to the opposite regulation of AKT activity, cell proliferation, and neural differentiation in cerebral organoids developed from UEC-derived hiPSCs. **(A)** A schematic illustration of the procedure for generating cerebral organoids that were treated with AKT- and PTEN-inhibitory small molecules. **(B)** The phosphorylated AKT substrate was detected by western blotting in the lysates of the cerebral organoids treated with afuresertib (an AKT inhibitor) and VO-OHpic (a PTEN inhibitor) at the indicated concentrations along with their development for 40 days. Con: DMSO-treated organoids. **(C)** The expression of MKI67 (a marker for proliferative cells) and SOX2 (a marker for neural progenitors) was detected by western blotting and immunofluorescence staining in the organoids treated with afuresertib (Af, 0.2 μM) and VO-OHpic (VO, 0.1 μM) along with their development for 40 days. *Upper panel:* Protein expression detected by western blotting. Con: DMSO-treated organoids. *Lower panel:* The immunofluorescence staining of MKI67 in control (DMSO-treated) and afuresertib-treated organoids. **(D)** The expression of multiple markers for neural differentiation was detected by immunofluorescence staining and western blotting in the inhibitor-treated organoids. *Left panel:* The immunofluorescence staining of markers for neural progenitors (SOX2 and HOPX), a pan-neuronal marker (TUBB3) and a marker for early-born neurons (BCL11B) in control and 0.2 μM afuresertib-treated organoids. *Right panel:* Protein expression of phosphorylated AKT, AKT, pan-neuronal markers (TUBB3 and RBFOX3) and markers for neural progenitors (HOPX and TBR2) detected by western blotting. Con: DMSO-treated organoids.

To further test how COs developed from UEC-derived hiPSCs may be affected by small molecules, we exposed UEC-derived hiPSC–developed COs to PTEN and AKT inhibitors (Figure 9A). VO-OHpic and afuresertib were used as representative inhibitors for PTEN and AKT, respectively. As expected, COs treated with VO-OHpic during their development showed the hyperactivation of AKT signaling, indicated by the enhanced phosphorylation of AKT substrates in the COs (Figure 9B). The upregulation of MKI67, SOX2, TBR2, and HOPX accompanied by the downregulation of TUBB3 and RBFOX3 was observed in the VO-OHpic-treated COs (Figures 9C,D). Moreover, the treatment of afuresertib led to the opposite regulation of these markers (Figures 9C,D). Our results indicate that UEC-derived hiPSC-developed COs can respond normally to the small molecule-mediated regulation of cell signaling that is strongly involved in differentiation, neurogenesis, and cerebral development.

## Discussion

Human iPSC-developed COs emerge as a powerful platform for paradigm-shifting research in neural development and pathogenesis. Although COs can be developed using hiPSCs reprogrammed from dermal fibroblasts, little is known about the capacity and features of hiPSCs reprogrammed from other somatic cells in CO formation. The optimization and characterization of COs developed from different types of hiPSCs is expected to contribute substantially to the basic science and translational research of the human brain. In this work, we have studied the development of COs using hiPSCs reprogrammed from urine sample-derived UECs.

Within 48 h after urine collection, proliferative human UECs can be obtained from urine samples through either centrifugation-based or filtration-based isolation (Figures 1C,D). Thus, this non-invasive method to obtain proliferative somatic cells from many individuals could be implemented easily by researchers or supporting staff with minimal training required. Although the subtype identity of the collected UECs that were reprogrammed and gave rise to hiPSCs from heterogenous UECs remains elusive, we were able to establish hiPSCs from the urine sample-derived UECs of multiple individuals with different ages and ethnic backgrounds. Nevertheless, the high heterogeneity of urine sample-derived UECs (Figure 1E) that could exist among different individuals may lead to the variable efficiency of cell reprogramming and hiPSC establishment in different samples. Further characterizing these UECs by single cell-based analysis in a future study is also expected to help address the cell-heterogeneity issue.

Because unique features may be associated with different hiPSCs reprogrammed from the same type of somatic cells using distinct methods (Schlaeger et al., 2015), we included UEC-derived hiPSCs generated using integrative and non-integrative systems to be tested in our studies. Despite the variations associated with sample donors and different methods for cell reprogramming, all the UEC-derived hiPSCs that we examined can develop into COs (Figure 3D and Supplementary Figure S1). Non-supervised clustering of samples based on their transcriptomic profiles revealed that the COs developed from UEC-derived hiPSCs and WA09 hESCs share highly similar features in gene expression after they pass the dynamic transition phase for commitment to the neural lineage (Figure 5A). Since the cortical cells found in WA09 hESC-developed COs use gene expression programs that resemble those of the human fetal neocortex to organize into cerebral cortex-like regions (Camp et al., 2015), the similarity between the gene expression of COs developed from UEC-derived hiPSCs and WA09 hESCs suggests that COs developed from UEC-derived hiPSCs may also recapitulate the developmental features of the human fetal neocortex. While transcriptomic features were similar in the day-80 COs developed from UEC-derived hiPSCs and WA09 hESCs, noticeable variations existed in the COs at earlier time points (Figure 5A). These variations may reflect different kinetics and efficiencies for neural commitment that are likely due to variable susceptibility to patterning signals in different cells and caused by their intrinsic variations (Figure 5C).

Though pluripotent, hiPSCs generated using distinct reprogramming methods and from different individuals’ UECs consistently encountered a problem with the inhibition of TGFβ and WNT signaling, but tolerated the inhibition of TGFβ and BMP signaling well for neural induction during CO formation (Supplementary Figure S1). Both approaches to induce neural differentiation were previously used for developing COs (Kadoshima et al., 2013; Bershteyn et al., 2017; Qian et al., 2018; Cederquist et al., 2019; Zhang et al., 2019) and tolerated well by WA09 hESCs in this study. Thus, the differential responses of UEC-derived hiPSCs and WA09 hESCs to the inhibition of TGFβ and WNT signaling during CO development would be irrelevant to the differences of individuals and cell reprogramming methods but rather reflecting molecular features that exist commonly in UEC-derived hiPSCs.

From gene expression profiling, we discovered the differential expression of multiple genes involved in the regulation of WNT, BMP, and TGFβ signaling pathways in UEC-derived hiPSCs and WA09 hESCs (Figures 5D,E). The hyperexpression of pro-apoptotic protein BAX and the hypoexpression of CHCHD2 were also found in all the UEC-derived hiPSC samples compared with WA09 hESCs (Figures 5F,G). Notably, CHCHD2 primes the potential of neuroectodermal differentiation in hPSCs and enhances the viability of their differentiated derivatives through modulating SMAD4 (Zhu et al., 2016). It has also been reported as an apoptosis inhibitor by promoting the binding of BCL-XL to BAX and limiting the activation of BAX (Liu et al., 2015). Although the knockdown of CHCHD2 appears to show a negligible effect on the neuroectoderm differentiation of hPSCs in monolayer culture (Markouli et al., 2019), in parallel with CHCHD2 hypoexpression in the UEC-derived hiPSCs, the attenuated interaction of BCL-XL and BAX was found in the aggregates of UEC-derived hiPSCs with suspension culture in the SB431542/IWR-1-containing medium (Figure 6C). Compared with LDN193189/A83-01-mediated neural induction, SB431542/IWR-1-mediated neural induction caused a higher propensity for apoptosis that was evidenced by the enhanced activation of CASP3 and BAX in the aggregates of UEC-derived hiPSCs (Figures 6A,B). With the concomitant treatment of Y-27632, a ROCK inhibitor with anti-apoptotic effects in hPSCs (Kurosawa, 2012), during SB431542/IWR-1-mediated neural induction (Supplementary Figure S4), some aggregates of UEC-derived hiPSCs can form healthy neuroepithelial spheres and continue to develop FOXG1-expressing COs after matrigel embedding (Figure 7B). Thus, the challenge of CO development initiated by the inhibition of TGFβ and WNT signaling in UEC-derived hiPSCs could be, at least partially, due to their apoptotic propensity resulted from the low expression of CHCHD2 and overcome by the treatment of anti-apoptosis agents.

Several genes that mediate epigenetic regulation (e.g., *MEG3*, JARID1D, and *HAT1*) or protein posttranslational modification (e.g., *DUSP1*) that are involved in developmental programs (Yang et al., 2012; Nagarajan et al., 2013; Yen et al., 2018) also showed distinct expression patterns in the UEC-derived hiPSCs (Figures 5F,G). Since the *JARID1D* gene is a Y-linked gene, the high expression of JARID1D in the male hiPSC lines that we analyzed, compared with WA09 hESCs established from a normal female blastocyst (Thomson et al., 1998), was expected. Although additional studies will be required to functionally determine the critical factors that mediate the different responses of UEC-derived hiPSCs and WA09 hESCs to the inhibition of TGFβ and WNT signaling during CO formation, our discoveries reveal that unique signaling-network features may frequently exist in UEC-derived hiPSCs and underlie their specific responses to different methods for the induction of CO development.

Given the knowledge that FGF2 and FGF19 signaling is critical for cerebellar development (Muguruma et al., 2015) and that the activation of WNT signaling promotes caudalization (Takata et al., 2017), we challenged COs developed from UEC-derived hiPSCs to potentially convert from a prosencephalic fate to a rhombencephalic fate through the activation of FGF2, FGF19, and WNT signaling together with the suppression of TGFβ signaling during CO formation. Regardless of the different methods initially used for neural induction, the upregulation of FOXG1 and the downregulation of several hindbrain markers (Figure 7) suggest that cells in the COs were driven to a prosencephalic fate. Upon exposure to conversion media that contain SB431542, FGF2, CHIR99021, and FGF19, the expression patterns of forebrain and hindbrain markers can be effectively reversed if the conversion treatment begins in the initial 2 weeks of CO development. Evidenced by the significant downregulation of POU5F1 and the upregulation of FOXG1 within the initial 2 weeks of regular CO development (Supplementary Figure S5), most cells in COs, if not all, have lost their cellular pluripotency and begun to commit to a prosencephalic fate at that time. Although we cannot fully exclude a possibility that the upregulation of hindbrain markers detected in the converted organoids may be due to the selective induction of a rhombencephalic program in residual pluripotent cells, the likelihood of having the residual pluripotent cells solely responsible for the reversal of forebrain and hindbrain marker expression in the converted organoids is low. Our findings support that cellular plasticity is present in COs developed from UEC-derived hiPSCs.

Cerebral organoids developed from UEC-derived hiPSCs continued developing and were vascularized after transplantation into the mouse brain (Figure 8B). The high frequency of having pigmented cells in the anterior implants but not in the posterior implants (Figures 8B,C) indicates that the development of the transplanted organoids may respond differentially to distinct microenvironments in the cerebral cortex and cerebellum. The hypoexpression of a forebrain marker and the upregulation of hindbrain markers (Figure 8D) observed in the posterior implants compared with the anterior implants further suggest that the cellular plasticity observed in the COs (Figure 7) may be leveraged to generate cell components of various brain regions based on the organoids patterned by unique niche signaling at different transplanted locations *in vivo*.

Similar to hyperactive AKT found in the COs developed from WIBR3 hESCs with PTEN mutation for modeling human macrocephaly (Li et al., 2017), our COs treated with a PTEN inhibitor during their development showed the enhanced phosphorylation of AKT substrates (Figure 9B). The upregulation of MKI67, SOX2, TBR2, and HOPX accompanied by the downregulation of TUBB3 and RBFOX3 found in the COs with PTEN mutation (Li et al., 2017) was also observed in the PTEN inhibitor-treated COs (Figures 9C,D). Moreover, the treatment of an AKT inhibitor led to the opposite regulation of these markers (Figures 9C,D). Since the treatment of other two AKT inhibitors, ipatasertib and MK-2206, also attenuates overexpansion as well as normalizes cell proliferation and neural gene expression in the PTEN-mutant COs (Li et al., 2017), the similar molecular features caused by the inactivation of PTEN and AKT in hESC-developed and UEC-derived hiPSC-developed COs suggest the suitability and potential use of UEC-derived hiPSCs in CO production for disease modeling and drug screening.

As a potential platform for investigating the development and pathogenesis of the human brain, the improved reproducibility and quality of COs are critical for neurological research based on the organoid system. Although the establishment of cells enriched in the cerebral cortex appears to emerge reproducibly from COs generated with different cell origins and growth environments (Velasco et al., 2019), the unique responses of UEC-derived hiPSCs to different protocols for CO development (Figure 3 and Supplementary Figure S1) indicate that variations among different cell origins can largely affect the efficacy of CO development under certain conditions. Thus, the production of COs may still require specific optimization for different hPSCs to permit robust results in each CO-based study.

Summarized in Supplementary Table S1, our work demonstrates the suitability, advantage, and potential challenge of using COs developed from UEC-derived hiPSCs to study cerebral development and pharmacological responses. COs generated with this unique stem cell source represent a valuable platform that could be easily adopted and further optimized to facilitate a variety of brain research.

## Materials and Methods

### Isolation of UECs From Urine Samples

The sterile sample cups were provided to each subject enrolled in the study for collection of minimal 250 ml of their midstream urine from one visit. The urine samples from eight adults (six males and two females) who were healthy at the time of sample collection were used in this study. The age of the sample donors ranged from 24 to 65 at the time of sample collection. For centrifugation-based isolation, UECs in around 250 ml of the urine samples from each individual were pelleted by centrifugation at 500 × *g* for 5 min in an ultracentrifuge (Beckman Coulter, Indianapolis, IN, United States). The cell pellets were resuspended in 25 ml of phosphate-buffered saline (PBS) containing 5% heat-inactivated fetal bovine serum (FBS), pelleted, resuspended in 2 ml of the urinary cell medium, and placed into a well of six-well cell culture plate. For filtration-based isolation, the urine samples were filtered through sterilized membrane filters made of polypropylene (Tisch Scientific, North Bend, OH, United States), nylon (Tisch Scientific, North Bend, OH, United States), and PC (Isopore^TM^; MilliporeSigma, Burlington, MA, United States) with 10 μm pore size in a reusable bottle top filtering device. The membranes were retrieved from the device and placed into a cell culture dish to directly culture UECs on each membrane. The detailed information relevant to the urinary cell medium is provided as part of Supplementary Materials and Methods.

### Cell Culture

WA09 hESCs were obtained from the WiCell Stem Cell Bank (WiCell Research Institute, Madison, WI, United States). UEC715i-501 hiPSCs were established using CytoTune Sendai Reprogramming Kit (Thermo Fisher Scientific, Carlsbad, CA, United States) and kindly provided by Dr. Jeanne Loring from The Scripps Research Institute. UEC001i-003, UEC001i-009, and UEC001i-010 hiPSCs were established through retroviral vector-mediated cell reprogramming by the method described previously (Wang et al., 2011). Except the use of TeSR-E8 medium (Stemcell Technologies, Vancouver, BC, Canada) and EDTA hPSC passaging solution (Thermo Fisher Scientific, Carlsbad, CA, United States) in this study, we generally followed the reported method (Wang et al., 2011) for culturing undifferentiated hPSCs in a feeder cell-free condition with 0.5 mg/ml growth factor-reduced matrigel (Corning, Tewksbury, MA, United States) in a DMEM/F12 medium for plate coating. All the hPSCs were routinely subcultured when cell density reached 80–90% in culture plates. The passage numbers of the hiPSCs used in our experiments were spanning across 25–70. Additional information relevant to cells used in this study is summarized in Supplementary Table S2. The experiments using hPSCs were performed in compliance with the guidelines and approval of the institutional biosafety committee at UNTHSC. All cells were periodically tested using the MycoAlert mycoplasma detection kit (Lonza, Walkersville, MD, United States) and free of mycoplasma.

### Karyotyping

Chromosomal analysis in UEC-derived hiPSCs by counting 20 metaphase spreads for each sample was performed using a contract research service provided by Molecular Diagnostic Services (San Diego, CA, United States).

### Cerebral Organoid Formation

The protocols for the development of COs from WA09 hESCs and UEC-derived hiPSCs are illustrated schematically in Figure 3. The detailed information relevant to the protocols is summarized in Supplementary Materials and Methods.

### Cerebellar Organoid Formation and Rhombencephalic Conversion in Cerebral Organoids

The generation of cerebellar organoids from UEC-derived hiPSCs was based on a protocol reported previously (Muguruma et al., 2015). For the conversion of COs from its prosencephalic fate into a rhombencephalic fate, the organoids were transferred into conversion media I, II, and III at the specific time points illustrated schematically in Supplementary Figure S4B. The detailed information relevant to the protocols is summarized in Supplementary Materials and Methods.

### Treatment of AKT and PTEN Inhibitors

Afuresertib was obtained from Cayman Chemical (Ann Arbor, MI, United States). VO-OHpic was obtained from Tocris (Minneapolis, MN, United States). The small-molecule inhibitors dissolved in DMSO as stock solutions were diluted in media and applied to COs during the time period illustrated schematically in Figure 9A.

### Immunofluorescence Staining

The general procedure for antibody-mediated fluorescence staining was previously described (Wang et al., 2011) and provided as part of Supplementary Materials and Methods. The detailed information of primary antibodies used in this study is summarized in Supplementary Table S3.

### Immunoprecipitation

The cell lysates were prepared using M-PER mammalian protein extraction reagent (Thermo Fisher Scientific, Carlsbad, CA, United States) containing EDTA-free protease inhibitor and phosphatase inhibitor cocktails (Millipore Sigma, St. Louis, MO, United States). Anti-BAX mouse IgG (MA5-14003; Thermo Fisher Scientific, Carlsbad, CA, United States) was pre-coated onto Dynabeads M-280 sheep anti-mouse IgG (Thermo Fisher Scientific, Carlsbad, CA, United States) at 4°C. The paramagnetic beads pre-coated with the antibody were mixed with cell lysates (80 μg total protein per lysate sample as an input) in PBS with the reaction volume of 400 μl at 4°C overnight, prior to the magnet-mediated separation of bead-bound proteins from the rest of the sample. The bead-bound proteins eluted off the beads were analyzed by immunoblotting with antibodies against specific targets.

### Immunoblotting

The general procedure for immunoblotting was described in a previously published report (Wang et al., 2008), except that cell lysates were prepared using M-PER mammalian protein extraction reagent (Thermo Fisher Scientific, Carlsbad, CA, United States) containing EDTA-free protease inhibitor and phosphatase inhibitor cocktails (Millipore Sigma, St. Louis, MO, United States). To detect multimerized BAX, cell lysates were prepared using a hypotonic buffer that contains 20 mM HEPES, 10 mM potassium chloride, and the protease inhibitor and phosphatase inhibitor cocktails. Bismaleimidohexane (Thermo Fisher Scientific, Carlsbad, CA, United States) was added into the lyates at the final concentration of 5 mM to stabilize oligomerized proteins through crosslinking. The detailed information of primary antibodies used in this study is summarized in Supplementary Table S3. HRP-conjugated secondary antibodies were from Jackson ImmunoResearch Laboratories (West Grove, PA, United States). For detecting targets in the bead-bound proteins from immunoprecipitation, TrueBlot HRP-conjugated secondary antibodies (Rockland Immunochemicals, Limerick, PA, United States) were used to specifically recognize the non-reduced form of primary antibodies.

### Gene Expression Analysis by qRT-PCR and Microarrays

The procedures for microarray analysis in this study are provided as part of Supplementary Materials and Methods. The test of cellular pluripotency based on the transcriptomic features of cell samples was performed using the PluriTest^1^ (Muller et al., 2011). Multiplex qRT-PCR was performed using cDNA generated from the RNA samples and Taqman assays for the *BMP4*, *BMPR1A*, *CTNNB1, LRP5, SMAD7, FOXG1, GBX2, HOXA2*, *HOXB4, POU5F1*, and *ACTB* (internal control) genes (assay ID# Hs00370078_m1, Hs01034913_g1, Hs00355049_m1, Hs00182031_m1, Hs00998193_m1, Hs01850784_s1, Hs00230965_m1, Hs00534579_m1, Hs00256884_m1, Hs00999632_g1, and Hs03023943_g1; Thermo Fisher Scientific, Carlsbad, CA, United States), according to the manufacturer’s instructions. The customized qRT-PCR arrays with primer sets from PrimePCR target-list panels for detection of the selected human WNT, TGFβ, and BMP signaling targets and reference genes (*ACTB* and *GAPDH*) were obtained from Bio-Rad (Hercules, CA, United States). The SYBR Green-based qRT-PCR reactions on the arrays were performed according to the manufacturer’s instructions.

### *In vivo* Studies

The procedures for organoid transplantation in this study are provided as part of Supplementary Materials and Methods.

### Statistical Analysis

Quantitative data reported in this work were reproducible in at least three biological replicates and presented as mean ± standard deviation. The significance of differences in comparisons was primarily determined by the two-tailed Student’s *t*-test for a two-group comparison or by the multivariate analysis of variance for testing variables in three or more groups.

### Study Approval

The collection of urine samples from human subjects and the isolation of UECs from the urine samples for experiments were performed in compliance with the guidelines and approval of the Institutional Review Board Committee at the University of North Texas Health Science Center (UNTHSC). All experimental procedures and protocols utilizing mice were approved by the Institutional Animal Care and Use Committee at UNTHSC.

## Data Availability Statement

The gene expression array data have been deposited and linked to an accession number GSE131562 in the Gene Expression Omnibus (GEO). Other data included in the article to demonstrate our findings are available from the corresponding author upon reasonable request. The biological samples used in this study may be distributed upon request and under institutional material transferring agreements or a licensing process.

## Ethics Statement

The studies involving human participants were reviewed and approved by the Institutional Review Board Committee at University of North Texas Health Science Center. The patients/participants provided their written informed consent to participate in this study. The animal study was reviewed and approved by the Institutional Animal Care and Use Committee at University of North Texas Health Science Center.

## Author Contributions

VL, AZ, and JH contributed to the study concept, data collection and analysis, and manuscript preparation. L-JY contributed to the data collection and analysis for the animal studies. Y-CW supervised the study, coordinated research efforts, and contributed to the study concept and design, data analysis, and manuscript writing. All authors reviewed and approved the manuscript.

## Conflict of Interest

The authors declare that the research was conducted in the absence of any commercial or financial relationships that could be construed as a potential conflict of interest.

## Footnotes

1 www.pluritest.org
