# Supplementary material for: Urine Sample-Derived Cerebral Organoids Suitable for Studying Neurodevelopment and Pharmacological Responses
Source: Front Cell Dev Biol. 2020 May 14;8:304. doi: 10.3389/fcell.2020.00304 (PMC7247822; doi:10.3389/fcell.2020.00304)
Supplement: Supplementary file 1 [file Data_Sheet_1.PDF]

Urine sample–derived cerebral organoids suitable for studying neurodevelopment and pharmacological responses

Victor J.T. Lin[1], Jiangnan Hu[1], Ashwini Zolekar[1], Liang-Jun Yan[1], Yu-Chieh Wang[1]*

[1] Department of Pharmaceutical Sciences, UNT System College of Pharmacy, University of North Texas Health Science Center, Fort Worth, TX, USA

*To whom correspondence should be addressed:

Yu-Chieh Wang, Ph.D.
Department of Pharmaceutical Sciences
The University of North Texas Health Science Center
3500 Camp Bowie Boulevard, RES-340F
Fort Worth, Texas 76107
Tel: 1-(817) 735-2944
Fax: 1-(817) 735-2603
Email: yu-chieh.wang@unthsc.edu

Running title: UEC-derived hiPSC-developed cerebral organoids

Keywords: urinary epithelial cells, hiPSCs, hESCs, neurodevelopment, cerebral organoids

Supplementary Information:

Materials and Methods

Isolation of UECs from urine samples

Basal urinary cell medium: complete KFSM/EPM (1:1)

Complete Keratinocyte-SFM (KFSM, 500 ml) was obtained from Thermo Fisher Scientific (Carlsbad, CA), containing 25 mg bovine pituitary extract (BPE), 2.5 µg epidermal growth factor (EGF), 0.03 µg/ml cholera toxin (MilliporeSigma, Burlington, MA) and penicillin-streptomycin (200 units/ml-200 µg/ml; Thermo Fisher Scientific, Carlsbad, CA). EPM (500 ml) was composed of 337.5 ml Dulbecco's modified eagle medium (DMEM) with high glucose (Thermo Fisher Scientific, Carlsbad, CA), 112.5 ml Ham's F-12 nutrient mix (Thermo Fisher Scientific, Carlsbad, CA), 50 ml heat-inactivated fetal bovine serum (FBS; Thermo Fisher Scientific, Carlsbad, CA).

Working urinary cell medium:

The working urinary cell medium contained 1x GlutaMax, 0.4 µg/ml hydrocortisone, 5 µg/ml insulin, 2.5 µg/ml transferrin, 20 µg/ml triiodythyronine, 10 *n*g/ml EGF, 1 µg/ml adenine. GlutaMax, insulin and EGF were obtained from Thermo Fisher Scientific (Carlsbad, CA); Hydrocortisone, insulin, transferrin, triiodythyronine and adenine were obtained from MilliporeSigma (Burlington, MA).

The isolated cells were maintained in the working medium with medium changing performed every three days. The 10x DMEM with high glucose for spiking into urine samples was prepared by dissolving a pack of the medium power (Thermo Fisher Scientific, Carlsbad, CA) for reconstitution of 1000 ml DMEM with high glucose in 100 ml sterile deionized water.

Cerebral organoid formation

hPSC medium:

The hPSC medium consisted of 20% KnockOut serum replacement, 1% non-essential amino acids (NEAA), 1% GlutaMax, 0.18% 2-mercaptoethanol, 25 *n*g/ml FGF2 in DMEM/F12. All the components were obtained from Thermo Fisher Scientific (Carlsbad, CA).

Neural induction medium:

Neural induction medium containing SB431542 and IWR-1 was composed of 10 µM SB431542 and 3 µM *endo*-IWR-1, 1% N2 supplement and 1 µg/ml heparin in the FGF2-deficient hPSC medium. Neural induction medium containing LDN193189 and A83-01 was composed of 100 *n*M LDN193189 and 500 *n*M A83-01 in the FGF2-deficient hPSC medium.

<u>Differentiation medium I, II and III:</u>

Differentiation medium I contained 1% N2 supplement, 0.025% insulin, 1% GlutaMax, 1% NEAA, penicillin-streptomycin (200 units/ml-200 µg/ml), 0.05% 2-mercaptoethanol in the Neurobasal™ medium. Differentiation medium II contained 1% N2 supplement, 2% B27 supplement without vitamin A, 0.025% insulin, 1% GlutaMax, 1% NEAA, penicillin-streptomycin (200 units/ml-200 µg/ml), 0.05% 2-mercaptoethanol in the Neurobasal™ medium. Differentiation medium III contained 1% N2 supplement, 2% B27 supplement with vitamin A, 0.025% insulin, 1% GlutaMax, 1% NEAA, penicillin-streptomycin (200 units/ml-200 µg/ml), 0.05% 2-mercaptoethanol in the Neurobasal™ medium. All the components of the differentiation media were obtained from Thermo Fisher Scientific (Carlsbad, CA).

Rhombencephalic conversion in cerebral organoids

<u>Conversion medium I, II and III:</u>

Conversion medium I contained 10 µM SB431542, 50 *n*g/ml FGF2, 4 µM CHIR99021 (Bio-Techne, Minneapolis, MN), and 0.5 *n*M retinoic acid (Bio-Techne, Minneapolis, MN) in the hPSC medium. Conversion medium II contained 100 *n*g/ml FGF19, 4 µM CHIR99021, and 0.5 *n*M retinoic acid in the FGF2-deficient hPSC medium. Conversion medium III contained 1% N2 supplement, 4 µM CHIR99021, 0.5 *n*M retinoic acid in the FGF2-deficient hPSC medium.

The time points for the use of these conversion media were annotated in the cerebral organoid protocols illustrated in Fig. S**4B**.

Immunofluorescence Staining

For the staining of pluripotency biomarkers in hPSCs and their differentiated derivatives, cells were plated into 24-well plates, fixed and permeabilized and incubated with primary antibodies against specific pluripotency biomarkers and fluorophore-conjugated secondary antibodies (Thermo Fisher Scientific, Carlsbad, CA). Organoid samples were fixed using 4% paraformaldehyde in PBS at at 4°C for overnight, rinsed with PBS, transferred into 15% sucrose

in PBS at 4°C for 48 hours, and transferred subsequently into 30% sucrose in PBS at 4°C for 48 hours. The samples were frozen embedding in a tissue-freezing matrix (O.C.T. compound; Thermo Fisher Scientific, Carlsbad, CA) at -80°C. For the staining of frozen sections of orgnoid and tissue samples, sections on slides were rinsed in phosphate-buffered saline (PBS) for three times to remove the tissue-freezing matrix, blocked with 5% donkey serum in PBS, and reacted with a primary antibody against a specific antigen at 4°C for overnight. After thorough washing with PBS containing 0.2% Tween-20 (PBST; Millipore Sigma, St. Louis, MO), the tissue sections were reacted with a flourescence-conjugated secondary antibody (Thermo Fisher Scientific, Carlsbad, CA) at room temperature for 1 hour, washed with PBST, and counterstained using a DAPI solution. If the biotinylated primary antibody was used, flourescence-conjugated streptavidin (Thermo Fisher Scientific, Carlsbad, CA) was subsequently used to reacted with the section. The stained organoid and tissue sections were mounted with cover slips using a Fluoromount-G medium (Southern Biotech , Birmingham, AL).

Gene Expression Analysis by qRT-PCR and Microarrays

Total RNA was isolated from cell samples using the mirVana miRNA Isolation Kit (Thermo Fisher Scientific, Carlsbad, CA). The quality of each RNA samples was determined using an Agilent 2200 Tape Station system (Agilent, Santa Clara, CA) for RNA integrity analysis. Samples with RIN^e numbers above 7 were chosen to move forward with global gene expression profiling. The iScript Reverse Transcription Supermix (Bio-Rad, Hercules, CA) was used to generate the cDNA of total RNA samples. The cDNA samples were used for qRT-PCR assays. Global gene expression profiling was performed using HT-12v4 Human Gene Expression Bead Chips and an iScan array scanning system (Illumina, Hayward, CA), according to the manufacturer's instructions. The gene expression array data have been deposited with links to an accession number GSE131562 in the Gene Expression Omnibus (GEO). Data were filtered for detection p value <0.01 in GenomeStudio (Illumina, Hayward, CA), and normalized using the LUMI package with RSN (Robust spline normalization) algorithm in R. Qlucore Omics Explorer 3.0 was used to perform differential gene expression analysis, hierarchical clustering, and plot generation. The ontology analysis of differentially expressed genes was performed using the PANTHER 14.1 (www.pantherdb.org).

In vivo Studies

Eight-week-old male NCG (NOD-*Prkdc*^{em26Cd52}*Il2rg*^{em26Cd22}/NjuCrl) mice (Charles River, Wilmington, MA) were group-housed under conditions of constant photoperiod (12 hours light: 12 hours dark) with *ad libitum* access to sterilized food and water. Cerebral organoids developed from UEC001i-009 hiPSCs were used in the animal study. Each isoflurane-anesthetized mouse was placed stably in a stereotactic frame with its body temperature maintained at 37 °C using a heating pad. Small incisions were made at the skin above the skull that covers the mouse cerebral cortex and cerebellum. Craniotomy to create a circular window of ~3mm diameter at the skull above each transplatation site was performed. Subsequently, the underlying dura mater was removed to access the brain tissue and create a small cavity to house the cerebral organoids at each transplantation region by aspiration with a blunt-end needle. The cavities were created unilaterally at the retrosplenial cortex and cerebellum in the same animal. Cerebral organoids without Matrigel embedding at the day 15 of development from the same batch of organoid production were inserted into the cavities. Each transplantation site received 3 organoids at similar size and without noticeable pigmentation. Each transplanted region was covered with a 3-mm cover slip that was mounted to skull using adhesive glue to create a cranial window. The surgical wound was closed with sutures. After the surgery, the mice were allowed to recover in a temperature-controlled cage before returning to their regular cages. The genneral condition of the mice was monitored on a daily basis throughout the entire study.

At terminal sacrifice, mice were anesthetized with a lethal dose of isoflurane and perfused transcardially with PBS (pH 7.2–7.5) followed by PBS containing 4% paraformaldehyde (PFA) freshly prepared. The whole animal brain was harvested and further fixed in PBS containing 4% PFA overnight. After fixation, the brain was transferred into 30% sucrose in PBS for 48 hours and subsequently frozen embedding in the O.C.T. compound at -80°C prior to cryosection.

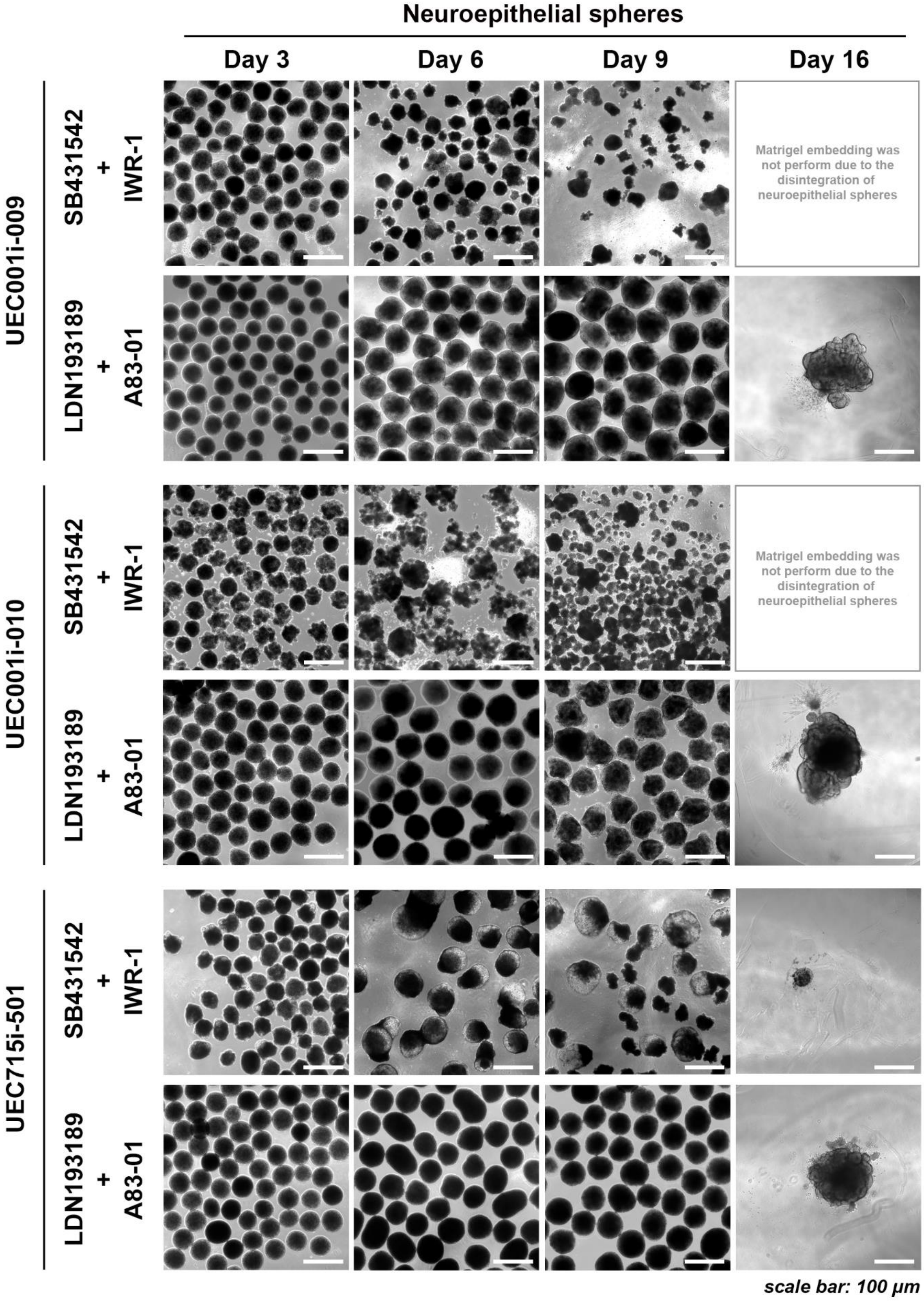
Neuroepithelial spheres
Day 3
Day 6
Day 9
Day 16
UEC001i-009
SB431542 + IWR-1
LDN193189 + A83-01
UEC001i-010
SB431542 + IWR-1
LDN193189 + A83-01
UEC715i-501
SB431542 + IWR-1
LDN193189 + A83-01
Matrigel embedding was not perform due to the disintegration of neuroepithelial spheres
Matrigel embedding was not perform due to the disintegration of neuroepithelial spheres
scale bar: 100 µm

Supplemental Figure 1. The differential responses of UEC-derived hiPSCs to different protocols for cerebral organoid formation. The morphological representations at the indicated time points of the cerebral organoids that were developed from UEC001i-009, UEC001i-010 and UEC715i-501 hiPSCs in response to inhibition of TGFβ and WNT signaling (SB431542 + IWR-1) or inhibition of TGFβ and BMP signaling (LDN193189 + A83-01). Within two weeks after the induction of neuroectodermal commitment by two different appraoches, disintegration occurred in cell spheres with the treatment of SB431542 and IWR-1. The organoids developed from the cells with the treatment of LDN193189 and A83-01 remained intact and continued to grow.

A

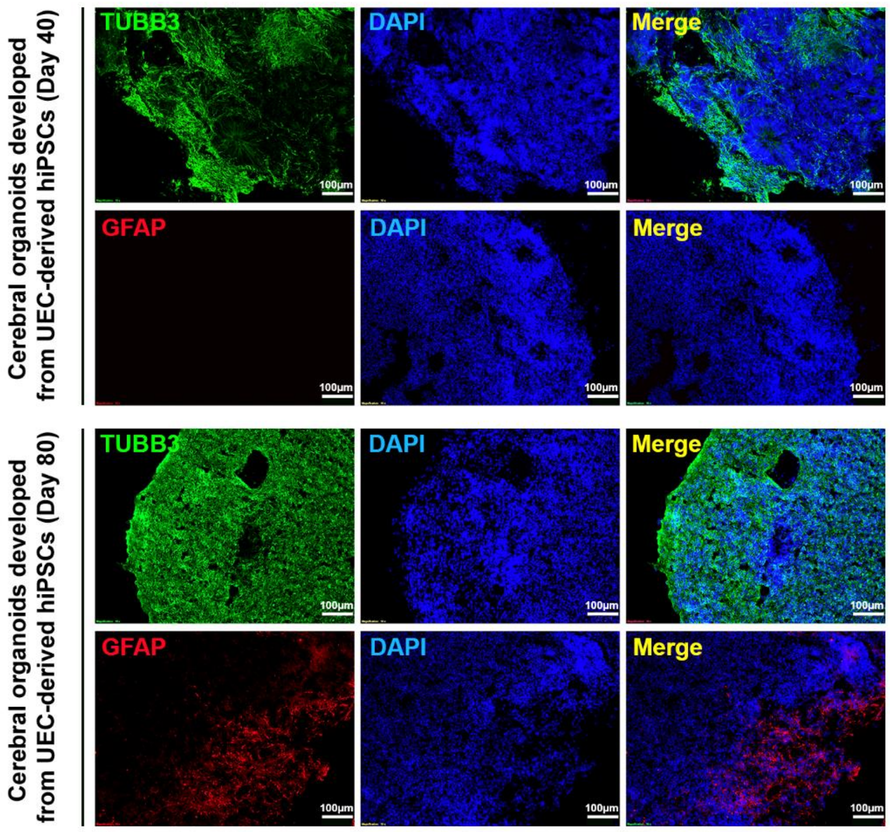
Cerebral organoids developed from UEC-derived hiPSCs (Day 40)
TUBB3
DAPI
Merge
GFAP
DAPI
Merge
Cerebral organoids developed from UEC-derived hiPSCs (Day 80)
TUBB3
DAPI
Merge
GFAP
DAPI
Merge
100µm

B

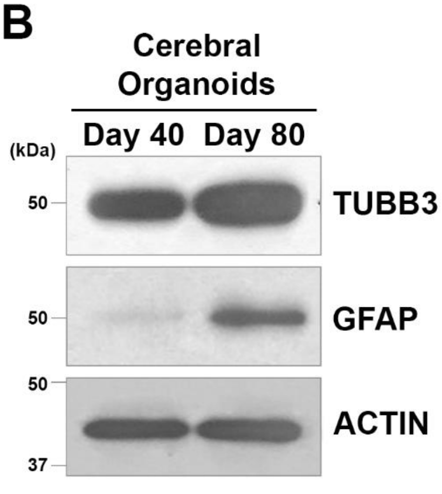
Cerebral Organoids
Day 40
Day 80
(kDa)
50
TUBB3
50
GFAP
50
ACTIN
37

Supplemental Figure 2. Neurogenesis followed by astrogliogenesis was observed in cerebral organoids developed from UEC-derived hiPSCs. (**A**) Immunofluorescence staining of TUBB3-positive neuronal cells and GFAP-positive astroglia were performed in the frozen sections of cerebral organoids collected on day 40 and day 80 of development from UEC001i-009 hiPSCs. Compared with the day-80 organoids, the day-40 organoids had none or a limited number of GFAP-positive cells. (**B**) The expression of TUBB3 and GFAP was also detected by western blotting in the day-40 and day-80 organoids. The low expression of GFAP detected in the day-40 organoids was concordant with the result from immunofluorescence staining.

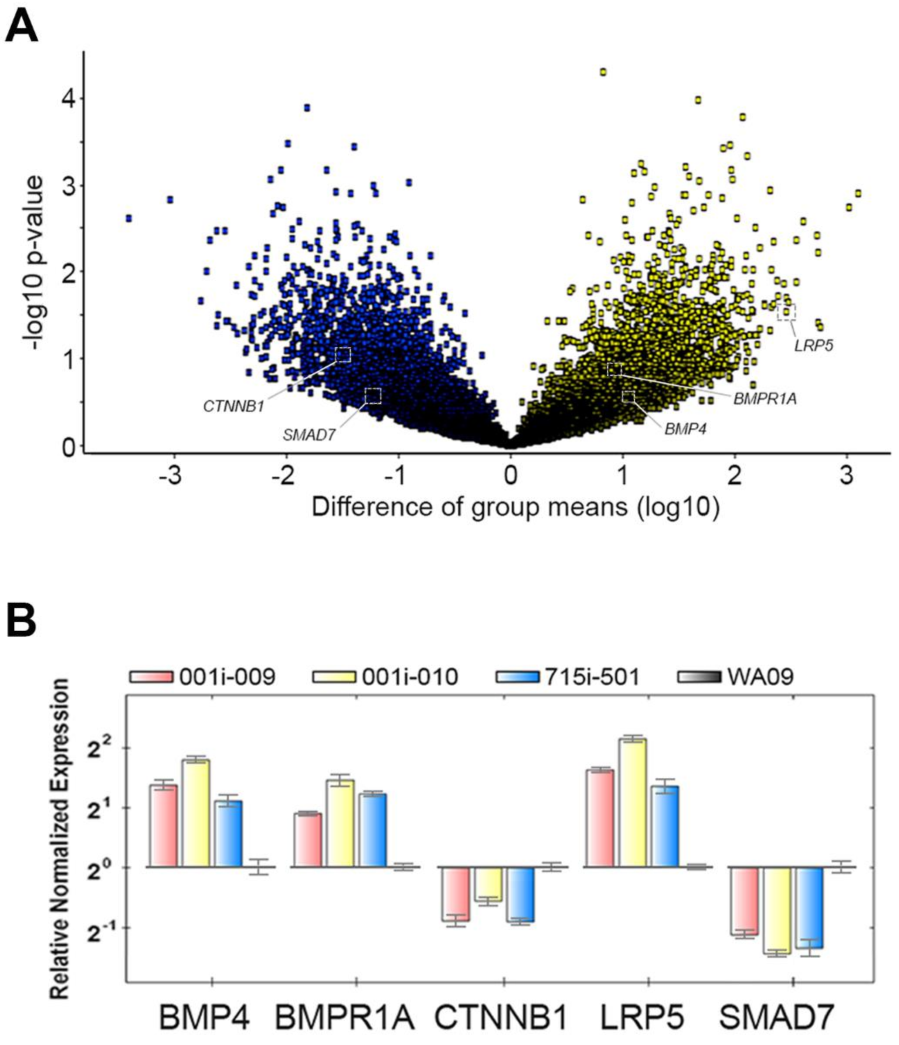
A
-log10 p-value
Difference of group means (log10)
-3
-2
-1
0
1
2
3
4
CTNNB1
SMAD7
LRP5
BMPR1A
BMP4
B
001i-009
001i-010
715i-501
WA09
Relative Normalized Expression
2^2
2^1
2^0
2^{-1}
BMP4
BMPR1A
CTNNB1
LRP5
SMAD7

Supplemental Figure 3. The distinct gene expression profiles in UEC-derived hiPSCs and WA09 hESCs were revealed by global gene expression profiling. (**A**) The volcano plot of expression difference (average expression in UEC-derived hiPSC samples – average expression in WA09 hESC samples) vs. significance. (**B**) The expression of the selected genes that were upregulated or downregulated in the analyzed UEC-derived hiPSC samples was validated by qRT-PCR analysis (*n*=3).

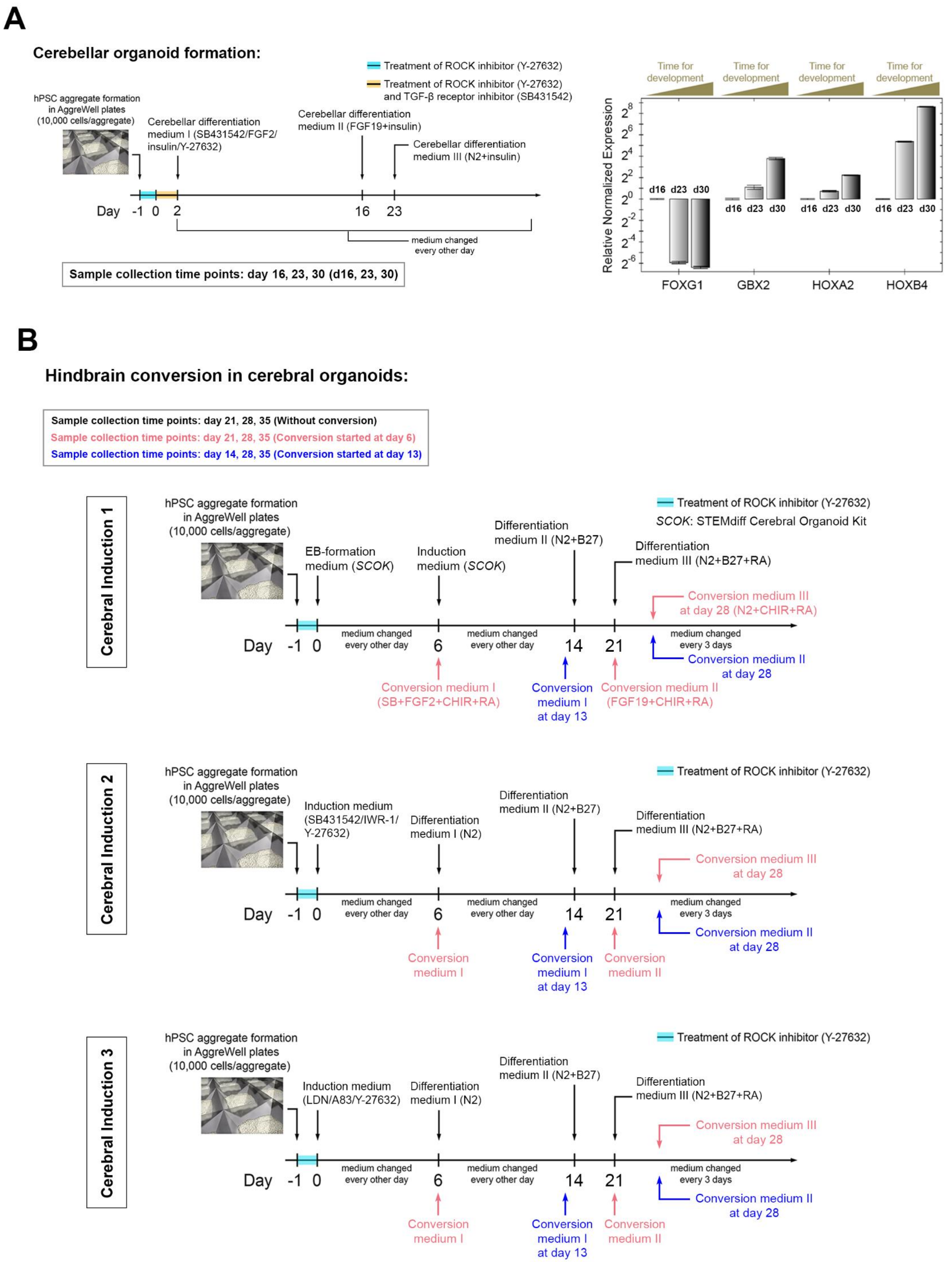
A
Cerebellar organoid formation:
Treatment of ROCK inhibitor (Y-27632)
Treatment of ROCK inhibitor (Y-27632) and TGF-β receptor inhibitor (SB431542)
hPSC aggregate formation in AggreWell plates (10,000 cells/aggregate)
Cerebellar differentiation medium I (SB431542/FGF2/ insulin/Y-27632)
Cerebellar differentiation medium II (FGF19+insulin)
Cerebellar differentiation medium III (N2+insulin)
Day -1 0 2 16 23
medium changed every other day
Sample collection time points: day 16, 23, 30 (d16, 23, 30)
Time for development
Relative Normalized Expression
d16 d23 d30
FOXG1
GBX2
HOXA2
HOXB4
B
Hindbrain conversion in cerebral organoids:
Sample collection time points: day 21, 28, 35 (Without conversion)
Sample collection time points: day 21, 28, 35 (Conversion started at day 6)
Sample collection time points: day 14, 28, 35 (Conversion started at day 13)
Cerebral Induction 1
hPSC aggregate formation in AggreWell plates (10,000 cells/aggregate)
Treatment of ROCK inhibitor (Y-27632)
SCOK: STEMdiff Cerebral Organoid Kit
EB-formation medium (SCOK)
Induction medium (SCOK)
Differentiation medium II (N2+B27)
Differentiation medium III (N2+B27+RA)
Conversion medium III at day 28 (N2+CHIR+RA)
Day -1 0 6 14 21
medium changed every other day
medium changed every 3 days
Conversion medium II at day 28
Conversion medium I (SB+FGF2+CHIR+RA)
Conversion medium I at day 13
Conversion medium II (FGF19+CHIR+RA)
Cerebral Induction 2
hPSC aggregate formation in AggreWell plates (10,000 cells/aggregate)
Treatment of ROCK inhibitor (Y-27632)
Induction medium (SB431542/IWR-1/ Y-27632)
Differentiation medium I (N2)
Differentiation medium II (N2+B27)
Differentiation medium III (N2+B27+RA)
Conversion medium III at day 28
Day -1 0 6 14 21
medium changed every other day
medium changed every 3 days
Conversion medium II at day 28
Conversion medium I
Conversion medium I at day 13
Conversion medium II
Cerebral Induction 3
hPSC aggregate formation in AggreWell plates (10,000 cells/aggregate)
Treatment of ROCK inhibitor (Y-27632)
Induction medium (LDN/A83/Y-27632)
Differentiation medium I (N2)
Differentiation medium II (N2+B27)
Differentiation medium III (N2+B27+RA)
Conversion medium III at day 28
Day -1 0 6 14 21
medium changed every other day
medium changed every 3 days
Conversion medium II at day 28
Conversion medium I
Conversion medium I at day 13
Conversion medium II

Supplemental Figure 4. The procedures to generate cerebellar organoids from hPSCs and to convert the prosencephalic fate of cerebral organoids into a rhombencephalic fate. (**A**) *Left panel:* A schematic illustration of the procedure used in this study to generate cerebellar organoids UECs from hPSCs. *Right panel:* The rhombencephalic features of cerebellar organoids developed from UEC001i-009 hiPSCs were indicated by the downregulation of FOXG1 and the upregulation of GBX2, HOXA2, and HOXB4 along with the organoid development. The expression of the *FOXG1*, *GBX2*, *HOXA2*, and *HOXB4* genes in organoids samples (*n*=4) collected at the indicated time points was determined using qRT-PCR analysis. (**B**) A schematic illustration of the procedures for rhombencephalic conversion in cerebral organoids generated by three different approaches of neural induction in hPSCs. Pink highlight: the procedure used for conversion started at day 6 of organoid development. Blue highlight: the procedure used for conversion started at day 13 of organoid development. SCOK: STEMdiff™ Cerebral Organoid Kit. SB: SB431542. CHIR: CHIR99021.

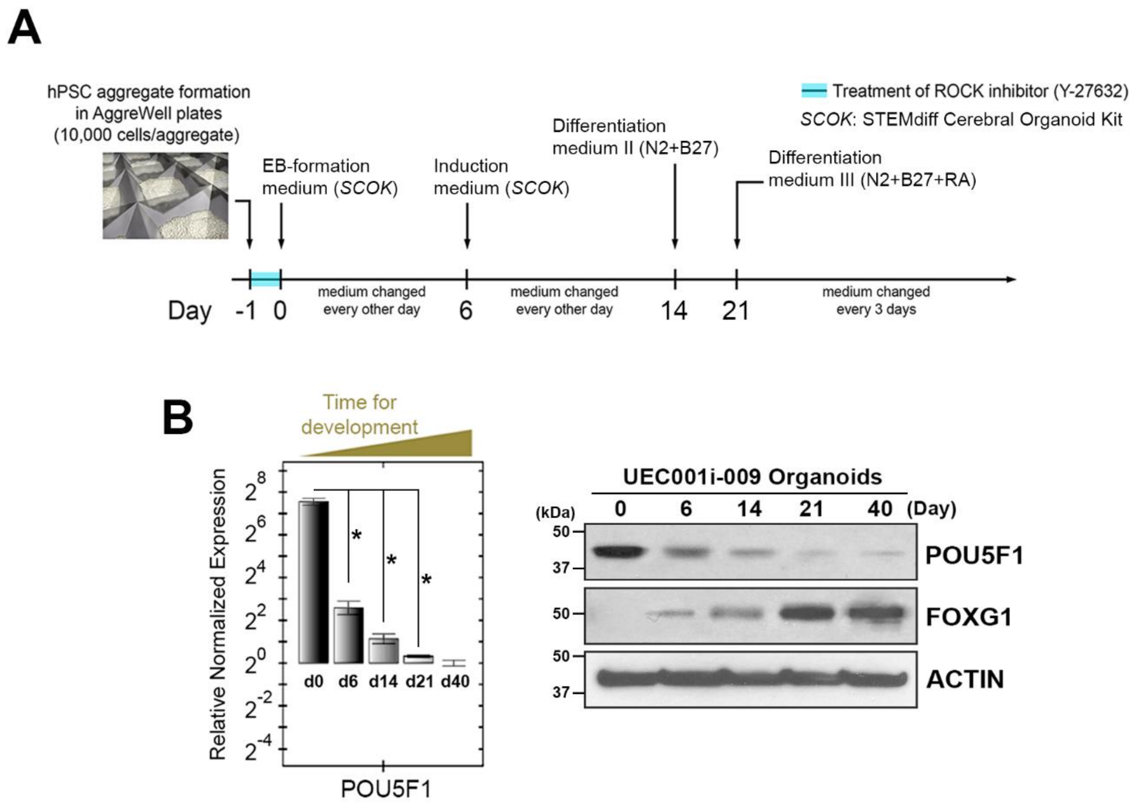
A
hPSC aggregate formation in AggreWell plates (10,000 cells/aggregate)
Treatment of ROCK inhibitor (Y-27632)
SCOK: STEMdiff Cerebral Organoid Kit
EB-formation medium (SCOK)
Induction medium (SCOK)
Differentiation medium II (N2+B27)
Differentiation medium III (N2+B27+RA)
Day -1 0 6 14 21
medium changed every other day
medium changed every other day
medium changed every 3 days
B
Time for development
Relative Normalized Expression
d0 d6 d14 d21 d40
POU5F1
UEC001i-009 Organoids
(kDa) 0 6 14 21 40 (Day)
POU5F1
FOXG1
ACTIN

Supplemental Figure 5. Cells in cerebral organoids developed from UEC-derived hiPSCs significantly lost their cellular pluripotency within two weeks at the beginning of organoid development. (**A**) A schematic illustration of the procedure for generating cerebral organoids that were used in the expression analysis of POU5F1 and FOXG1. (**B**) The expression of POU5F1 and FOXG1 was detected by qRT-PCR (*left panel*) and western blotting (*right panel*) in organoid samples collected at the indicated time points (n=3, *p<0.05, t-test).

**Supplemental Table S1. Major similarities and differences between ESC- and UEC-derived COs**	
**ESC-derived COs**	**UEC-derived COs**
**The challenge of hPSC sources**	
High	Low to moderate
**The variation of neuroepithelial sphere formation in response to different protocols**	
Unnoticed to low	Clearly noticed variations among protocols
**Cell propensity for apoptosis during development**	
Low	Moderate to high (could be overcome by protocol optimization)
**Cell plasticity permitting conversion between prosencephalic and rhombencephalic fates**	
Present	Present
**The overall capacity of normal development**	
High	Good
**Utility in studies required different genetic backgrounds and/or a personalized setting**	
Limited	High

Supplemental Table S2. The list of cultured cells used in the study		
**Sample Name**	**Registry Name[a]**	**Note[b]**
**Human embryonic stem cells**		
WA09	WA09	Obtained from the WiCell Stem Cell Bank; feeder cell-free culture on Matrigel, passaged using EDTA-based hPSC passaging solution
**Induced pluripotent stem cells from human Urinary Epithelial Cells (UECs)**		
UEC715i-501	N/A	Sendai virus-mediated reprogramming in the UECs of a healthy male donor (#715); provided by Dr. Jeanne Loring from The Scripps Research Institute; feeder cell-free culture on Matrigel, passaged using EDTA-based hPSC passaging solution
UEC001i-003	N/A	Retrovirus-mediated reprogramming in the UECs of a healthy male donor (#001); feeder cell-free culture on Matrigel, passaged using EDTA-based hPSC passaging solution
UEC001i-009	N/A	Retrovirus-mediated reprogramming in the UECs of a healthy male donor (#001); feeder cell-free culture on Matrigel, passaged using EDTA-based hPSC passaging solution
UEC001i-010	N/A	Retrovirus-mediated reprogramming in the UECs of a healthy male donor (#001); feeder cell-free culture on Matrigel, passaged using EDTA-based hPSC passaging solution

a. Name of cell line submitted to University of Massachusetts (UMass) International Stem Cell Registry

b. Somatic cell type, reprogramming method, culture condition, source of cells

N/A: not applicable

Supplemental Table S3. The list of primary antibodies and lectin used in the study		
**Antibody/Lectin Name**	**Catalog Number**	**Sources**
**Antibodies used in IHC or fluorescence staining**		
TRA-1-81	09-0011	Stemgent
POU5F1	2840	Cell Signaling Technology
NANOG	MABD24	Millipore Sigma
TUBB3	MRB-435P	Biolegend (formerly Covance)
Smooth Muscle Actin (SMA)	MAB1420	R&D Systems
SOX17	AF1924	R&D Systems
CSPG4	ab83178	Abcam
Brachyury	sc-17745	Santa Cruz Biotechnology
SOX2	AB5603	Millipore Sigma
TBR2	AB2283	Millipore Sigma
TBR1	ab31940	Abcam
Phospho-VIM	D076-3	MBL International
SATB2	ab34735	Abcam
BCL11B (CTIP2)	ab18465	Abcam
HOPX	HPA030180	Millipore Sigma
DCX2	sc-8066	Santa Cruz Biotechnology
GFAP	ab7260	Abcam
OTX2	MAB10289	Abnova
FOXG1	ab18259	Abcam
Human Nuclear Antigen	MAB1281B	Millipore Sigma
GBX2	114-182	Abnova
HOXA2	PA5-68986	Thermo Fisher Scientific
HOXB4	PA5-23366	Thermo Fisher Scientific
MKI67	ab16667	Abcam
**Antibodies used in immunoblotting**		
GFAP	ab7260	Abcam
TUBB3	MRB-435P	Biolegend (formerly Covance)
FOXG1	ab18259	Abcam
BAX	2772	Cell Signaling Technology
BCL-XL	2764	Cell Signaling Technology
CASP3	14220	Cell Signaling Technology
JARID1D	A301-751A	Bethyl Laboratories
ACTIN	08691001	MP Biomedicals
POU5F1	2840	Cell Signaling Technology
NANOG	MABD24	Millipore Sigma
CHCHD2	HPA027407	Millipore Sigma
DUSP1	HPA069577	Millipore Sigma
Phospho-AKT Substrate	9614	Cell Signaling Technology
Phospho-AKT (Ser473)	4060	Cell Signaling Technology
AKT	2920	Cell Signaling Technology
TBR2	AB2283	Millipore Sigma
RBFOX3	MAB377	Cell Signaling Technology
HOPX	PA5-49449	Thermo Fisher Scientific
SOX2	3579	Cell Signaling Technology
MKI67	ab16667	Abcam
**Lectin used in fluorescence staining**		
UEA-I	FL-1061	Vector Laboratories
